# Molecular insights and translational opportunities to enhance heat tolerance in rice

**DOI:** 10.1002/tpg2.70281

**Published:** 2026-07-17

**Authors:** Prabhat Rana, Chanderkant Chaudhary, Rajat Pruthi, Mansi Sharma, Bhupinderjeet Singh, Ravi Kiran Reddy Kondi, Mallesham Bulle, Prasanta K. Subudhi

**Affiliations:** ^1^ School of Plant, Environmental and Soil Sciences Louisiana State University Agricultural Center Baton Rouge Louisiana USA; ^2^ Department of Horticulture Oregon State University Corvallis Oregon USA; ^3^ College of Earth, Ocean, and Atmospheric Sciences Oregon State University Corvallis Oregon USA

## Abstract

Heat stress is an increasingly serious threat to rice (*Oryza sativa L*.) productivity, yet the genetic and regulatory architecture underlying thermotolerance remain poorly resolved and fragmented across studies. Earlier research focused on individual pathways or specific developmental stages; however, recent advances now support an integrated understanding of heat stress adaptation in rice. This review synthesizes emerging insights into molecular physiology, regulatory signaling, epigenetic memory, and genome‐scale variation associated with thermotolerance. We highlight the interconnected roles of calcium reactive oxygen species signaling, heat shock transcription factor networks, translational regulation, and chromatin‐based stress memory in shaping reproductive‐stage tolerance and maintaining grain quality under elevated temperatures. The review also emphasizes the value of pangenome analyses and structural variant discovery for identifying heat‐responsive genes and regulatory elements absent from single‐reference genomes. In addition, genome‐wide association studies, haplotype‐based breeding, genomic selection, and CRISPR (Clustered Regularly Interspaced Short Palindromic Repeats)‐based genome editing are discussed as promising approaches for functional validation and deployment of favorable alleles controlling polygenic heat resilience. Despite these advances, several challenges continue to hinder translation into breeding‐ready outcomes, including limited field‐based validation of candidate genes, poor integration of multi‐omics datasets into predictive breeding frameworks, and insufficient understanding of reproductive‐stage regulatory networks. Furthermore, genotype × environment interactions, together with trade‐offs among yield, grain quality, and stress resilience, strongly influence the stability and transferability of thermotolerance traits across diverse agroecological environments. By integrating mechanistic insights with genome‐scale diversity and predictive breeding tools, this review outlines a genomics‐enabled roadmap for developing heat‐resilient rice cultivars under intensifying global warming and supporting sustainable global rice production.

## Plain Language Summary

Higher temperatures are increasingly threatening rice production, particularly during flowering, when heat can cause severe yield losses and poor grain quality. To address this challenge, we reviewed recent advances in understanding how rice plants sense, respond to, and survive heat stress. We bring together new insights on stress signaling, protection of cellular proteins, regulation of grain filling, and epigenetic stress memory, and connect them with modern genome‐based breeding tools. Our review highlights emerging opportunities from pangenome analysis, haplotype‐based breeding, genomic selection, and gene editing to identify and deploy useful heat‐tolerant genes more efficiently. The key takeaway is that integrating molecular discoveries with advanced genomics provides a practical pathway to develop climate‐resilient rice varieties capable of sustaining yield and quality under rising global temperatures.

**Abbreviations**
ABA: abscisic acid
APX: ascorbate peroxidase
ASF1: anti‐silencing function 1
BRs: brassinosteroids
CaM: Ca^2^^+^/calmodulin
CAT: catalase
CDPKs: Ca^2^^+^‐dependent protein kinases
CMIP6: coupled model intercomparison project phase 6
CNGC: cyclic nucleotide‐gated channels
CONUS: the contiguous United States
CSD: Cu/Zn superoxide dismutases
CTKs: cytokinins
DREB2A: dehydration‐responsive element binding protein 2A
*FGT1*
: *Forgetter1*
GCM: global climate models
GS: genomic selection
GWAS: genome‐wide association studies
HSEs: heat shock elements
HSFs: heat shock transcription factors
HSPs: heat shock proteins
IP_3_
: inositol trisphosphate
IPT: isopentenyl transferase
IRE1: inositol‐requiring enzyme 1
LOCA: localized constructed analogs
MBF1c: multiprotein bridging factor 1C
MDA: malondialdehyde
OEC: oxygen‐evolving complex
OsHCI1: heat and cold induced 1
OsHIRP1: heat‐induced RING finger protein 1
OsHTAS: heat tolerance associated small protein
POD: peroxidase
PSII: photosystem II
QTLs: quantitative trait loci
RBOHs: respiratory burst oxidase homologs
ROS: reactive oxygen species
Rubisco: ribulose‐1,5‐bisphosphate carboxylase/oxygenase
SA: salicylic acid
sHSPs: small heat shock proteins
SNPs: single nucleotide polymorphisms
SOD: superoxide dismutase
SPL: squamosa‐promoter binding protein‐like
SVs: structural variants
TAS1: trans‐acting SiRNA precursor 1
TEs: transposable elements
TFs: transcription factors
Tmax: daily maximum temperature
TOGR1: thermo‐sensitive Obg‐like GTPase 1
TT1: thermo tolerance 1
UPR: unfolded protein response

## 1 INTRODUCTION

Rising temperatures constitute one of the most consequential environmental stressors influencing crop growth, development, and geographical distribution (B. Li, Gao, et al., 2018). Population growth and industrialization have increased greenhouse gas emissions, accelerating global warming and challenging agricultural systems globally (Quint et al., 2016). A projected increase of 1°C in global mean temperature could reduce crop yield by 3.4% for rice (C. Zhao et al., 2017). Furthermore, rice is expected to face at least 5 days of extreme heat stress during its reproductive stage, with temperatures surpassing critical physiological thresholds (Gourdji et al., 2013). Compounding these challenges, the Food and Agriculture Organization, estimates that feeding a projected global population of 9 billion will require more than a 70% increase in food production (Bita & Gerats, 2013).

Rice (*Oryza sativa* L.) is a globally important cereal crop that has substantial water requirements throughout its growth cycle (X. Wu et al., 2009). Elevated temperatures are closely linked to reduced crop productivity, as heat stress disrupts key physiological processes such as photosynthesis, water‐use efficiency, reproductive development, pollen viability, seed set, and grain quality (Hatfield & Prueger, 2015). In rice, the reproductive stage is particularly heat‐sensitive, with high day (>35°C) and night (27°C–30°C) temperatures impairing pollen development, spikelet fertility, and grain formation (Prasad et al., 2006). Heat stress during microsporogenesis and anthesis mainly affects male reproductive functions by reducing pollen viability and anther dehiscence, leading to spikelet sterility (Matsui et al., 2001; S. Yoshida et al., 1981). While female reproductive organs are comparatively heat tolerant, post‐fertilization stages such as grain filling are highly susceptible to heat stress, causing grain chalkiness and significant losses in yield and milling quality (Lyman et al., 2013). Addressing these challenges requires a comprehensive understanding of the morphological, physiological, and molecular mechanisms underlying heat stress adaptation (Sailaja et al., 2015).

This review synthesizes current understanding of heat stress tolerance mechanisms in rice and integrates recent advances in genomics‐enabled breeding. It highlights the contributions of genome‐wide association studies (GWAS), pangenome analyses, and genomic selection (GS) in identifying alleles and structural variants (SVs) underlying reproductive stability and grain quality under heat stress. Collectively, these approaches enhance discovery of causal genes and accelerate the development of heat resilient rice cultivars for warming climates.

## 2 STATUS OF GLOBAL WARMING AND IMPLICATIONS FOR FOOD SECURITY

Global warming is intensifying heat stress across major rice‐growing regions, especially in South and Southeast Asia and sub‐Saharan Africa, where most of the world's rice is produced and consumed (FAO, 2024; Legg, 2021; Muthayya et al., 2014). The IPCC projects that heatwaves will become more frequent, longer, and more severe, with substantial increases in growing‐season days above 35°C by mid‐century (Legg, 2021; Seneviratne et al., 2021; Serdeczny et al., 2017). Because rice is highly sensitive to heat during reproduction, even brief exposure above 35°C can sharply reduce yield; a 1°C rise in global mean temperature is estimated to lower rice yield by 3.4%, and temperatures of ≥35°C–38°C during anthesis can cause spikelet sterility exceeding 50% under severe stress (Jagadish et al., 2007; Prasad et al., 2006; C. Zhao et al., 2017). Heat impacts also vary across production systems and environments. Rainfed systems are especially vulnerable because combined heat and water stress increases yield instability, while irrigated systems can buffer some stress but remain highly sensitive during flowering and grain filling (Jagadish et al., 2007; Wassmann et al., 2009). Together, these regional and agroecological differences highlight the urgent need for context‐specific strategies to improve heat tolerance in rice.

Daily maximum temperature (Tmax) projections for the contiguous United States (CONUS) were derived from the statistically downscaled coupled model intercomparison project phase 6 (CMIP6) LOCA2 dataset (where LOCA is localized constructed analogs) (Pierce et al., 2023). LOCA2 applies an updated LOCA statistical downscaling approach to CMIP6 global climate models (GCMs) using a modified Livneh observational dataset for training (Pierce et al., 2021, 2023). We analyzed 23 CMIP6 GCMs for the historical period (through 2014) and under two future emissions scenarios, SSP2‐4.5 and SSP5‐8.5. All analyses were conducted over CONUS land grid cells at the native LOCA2 resolution of 0.0625° (∼6 km).

To focus on major US rice‐producing areas, we used USDA National Agricultural Statistics Service county‐level rice harvested acreage data to identify rice‐producing counties (USDA NASS, 2023). Within each major rice‐producing state Arkansas, California, Louisiana, Mississippi, Missouri, and Texas. We ranked counties by harvested acreage and selected the smallest set accounting for at least 95% of total state rice acreage. Climate metrics were then calculated only for LOCA2 grid cells within these selected counties.

Because the biological and agronomic literature synthesized here focuses mainly on rice systems in South and Southeast Asia and the southern United States, we extended the climate analysis beyond the United States to major rice‐producing regions in India, China, and the Philippines, which together account for nearly 57% of global rice production (USDA FAS, 2025). To ensure methodological consistency, we applied the same heat‐exposure metric using comparable CMIP6‐based datasets. For regions outside the CONUS, we used the NASA NEX‐GDDP‐CMIP6 dataset, which provides global daily downscaled and bias‐corrected CMIP6 projections at 0.25° (∼25 km) resolution (Thrasher et al., 2012, 2021). This dataset includes ScenarioMIP projections developed for CMIP6 and IPCC AR6 and uses quantile mapping for bias correction of daily climate outputs. For India and the Philippines, administrative boundary shapefiles were used to aggregate grid‐cell values to major rice‐growing states or provinces. Selected regions included Punjab, Uttar Pradesh, and West Bengal in India (Khush, 2005); Hunan, Jiangxi, Heilongjiang, Hubei, Anhui, Jiangsu, Guangdong, Guangxi, Sichuan, and Yunnan in China (L. Tang et al., 2022); and Nueva Ecija, Isabela, Pangasinan, Cagayan, and Iloilo in the Philippines (Bartelet et al., 2025).

Heat exposure was quantified as the annual number of days during the primary growing season (April–September) with Tmax ≥ 37°C (Prasad et al., 2006). For each grid cell, qualifying days were counted annually and averaged to generate climatological means for 1971–2000, 2025–2040, and 2040–2055. The 1971–2000 period served as the historical baseline, and anomalies for future periods were calculated for each model relative to this baseline. Metrics were then aggregated to state and regional scales using area‐weighted means. Multi‐model ensemble means were computed across all GCMs, and inter‐model spread was used to characterize projection uncertainty. Although this metric provides a robust and interpretable indicator of large‐scale extreme heat exposure, it does not distinguish between acute heat shocks and prolonged chronic heat stress, which have different physiological and yield effects. Brief exposure to temperatures ≥35°C–38°C during anthesis can cause immediate spikelet sterility through reduced pollen viability and impaired anther dehiscence (Jagadish et al., 2007; Matsui et al., 2001), whereas prolonged moderately elevated temperatures during grain filling can disrupt metabolism, reduce starch biosynthesis, and increase grain chalkiness (Folsom et al., 2014; Wada et al., 2015). These contrasts indicate that heat stress impacts depend not only on threshold exceedance but also on stress duration and developmental timing. Future studies would benefit from incorporating additional metrics, including heatwave duration, consecutive hot days, and thermal time above critical thresholds, to better capture the mechanisms of heat stress.

**FIGURE 1:**
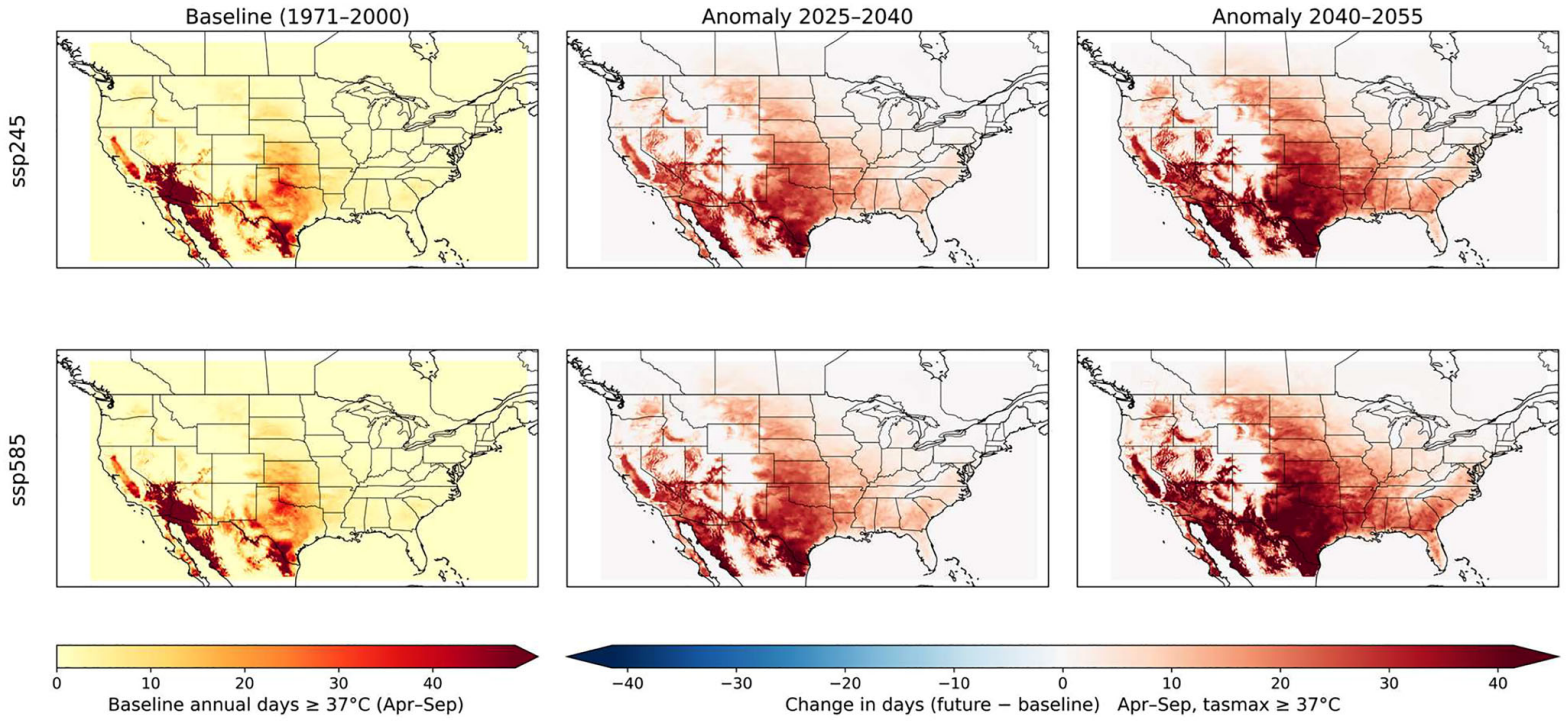
Spatial distribution of mean April–September (Apr–Sep) days with maximum temperature (Tmax) ≥ 37°C across the contiguous United States under two emissions scenarios. The left column shows the historical baseline (1971–2000). The middle and right columns show projected anomalies (future minus baseline) for 2025–2040 and 2040–2055, respectively. The top row corresponds to SSP2‐4.5 and the bottom row to SSP5‐8.5. Colors indicate the annual number of hot days (baseline panels) and changes in the number of hot days (anomaly panels). Positive values (red shading) represent increases relative to the historical period.

During the historical baseline period (1971–2000), the CONUS area‐weighted mean number of April–September hot days (Tmax ≥ 37°C) was 4.26 days per season, with the highest frequencies concentrated in the southern United States and interior California (Figure 1). Both SSP2‐4.5 and SSP5‐8.5 project pronounced and spatially extensive intensification of extreme heat exposure.

**FIGURE 2:**
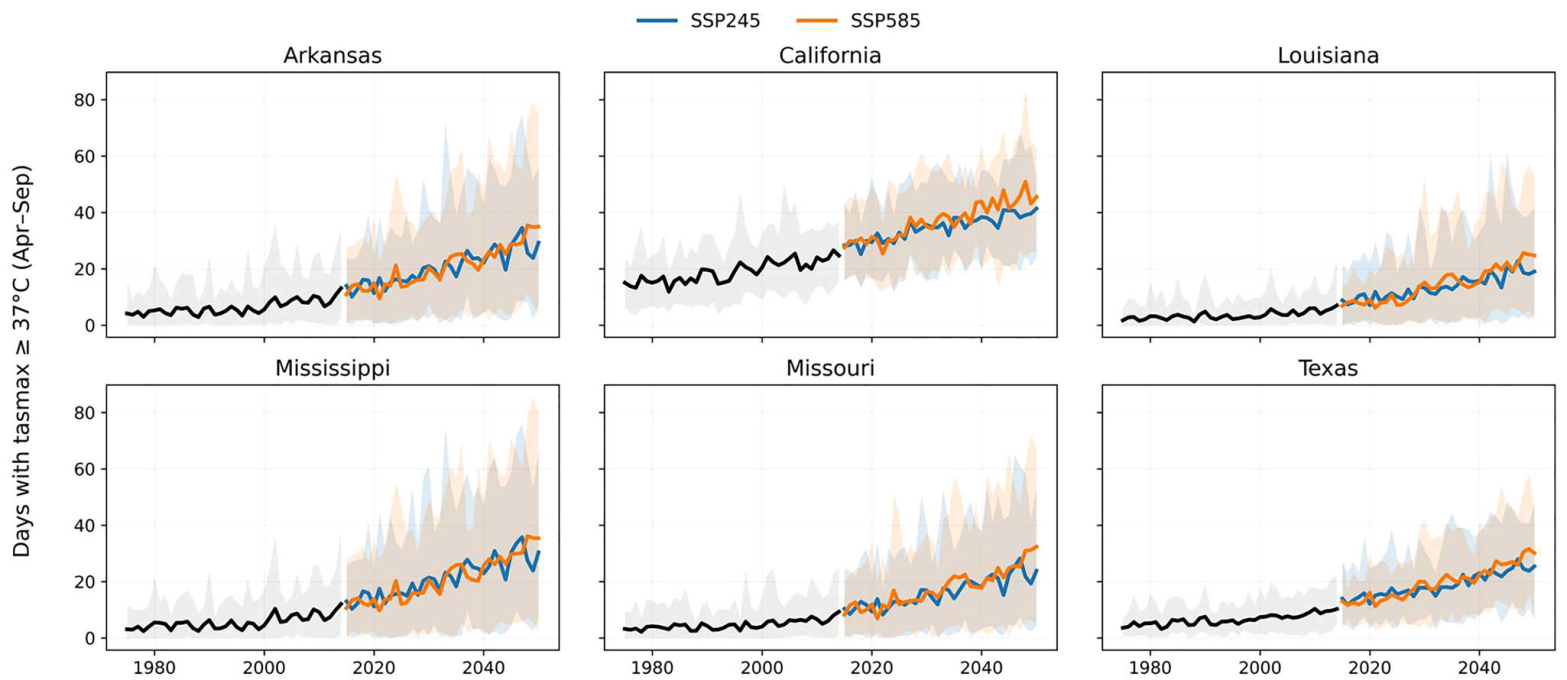
Time series of projected April–September (Apr–Sep) days with maximum temperature (Tmax) ≥ 37°C for major rice‐producing counties in six US states: Arkansas, California, Louisiana, Mississippi, Missouri, and Texas. For each state, the analysis includes the shortlisted counties that collectively account for approximately 95% of the state's rice harvested acreage based on USDA National Agricultural Statistics Service (NASS) county‐level statistics. The historical period is shown in black, followed by future projections under SSP2‐4.5 (blue) and SSP5‐8.5 (orange). Solid lines represent the multi‐model mean across GCMs, and shaded bands indicate inter‐model spread.

By 2025–2040, the CONUS mean increases by 5.40 days under SSP2‑4.5 and 5.95 days under SSP5‑8.5, and by 2040–2055 the increases reach 7.75 days (SSP2‑4.5) and 9.62 days (SSP5‑8.5), making hot days roughly 1.8–2.3 times more frequent than during the baseline. Under SSP5‑8.5, 65.7% of CONUS experiences an increase in ≥37°C days by 2040–2055, with 30.8% of the region exceeding 10 additional days and 20.4% exceeding 20 additional days per season. The most pronounced intensification occurs across Texas, the southern Great Plains, the lower Mississippi Valley, and interior California. State‐level time series for the shortlisted rice‐producing counties reveal a marked amplification of growing‐season extreme heat exposure across all major US rice‐producing regions (Figure 2). During the historical baseline period (1971–2000), baseline hot‐day frequencies ranged from 2.8 days per season in Louisiana and 3.8 days in Missouri to 16.9 days in California. Under SSP5‐8.5, by 2040–2050, projected hot‐day frequencies rise sharply to 44.3 days per season in California (+27.5 days), 30.1 days in Mississippi (+25.9 days), 29.0 days in Arkansas (+24.1 days), 26.7 days in Texas (+21.3 days), 25.1 days in Missouri (+21.3 days), and 21.3 days in Louisiana (+18.5 days). Although increases under SSP2‐4.5 are consistently smaller, they remain substantial, ranging from approximately +15.3 days in Louisiana to +23.8 days in Mississippi by mid‐century. Across all major rice‐producing regions, SSP5‐8.5 produces greater heat exposure than SSP2‐4.5, with divergence between emissions pathways becoming increasingly pronounced after the mid‐2030s.

**FIGURE 3:**
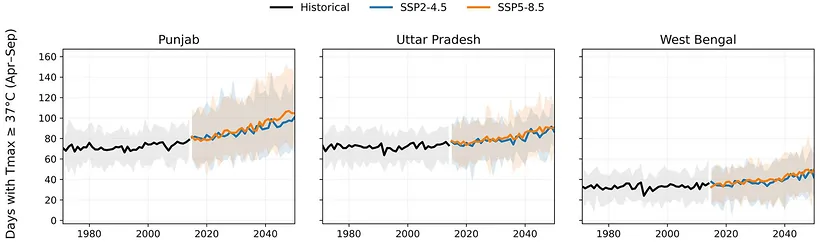
Time series of projected April–September (Apr–Sep) days with maximum temperature (Tmax) ≥ 37°C for major rice‐producing districts in the Indian states of Punjab, West Bengal, and Uttar Pradesh. The analysis includes the shortlisted districts that collectively account for most of the rice cultivated area in each state based on regional agricultural statistics. The historical period is shown in black, followed by future projections under SSP2‐4.5 (blue) and SSP5‐8.5 (orange). Solid lines represent the multi‐model mean across global climate models (GCMs), and shaded bands indicate inter‐model spread.

Across major rice‐growing states in India, the number of April–September hot days (Tmax ≥ 37°C) increases sharply under both SSP2‐4.5 and SSP5‐8.5 (Figure 3). Relative to a historical mean of 58.1 days per season (1971–2000), ensemble averages rise to 67.5 and 69.6 days by 2025–2040, and to 76.2 and 80.4 days by 2040–2055 under SSP2‐4.5 and SSP5‐8.5, respectively. In contrast, extreme heat exposure remains minimal across the selected rice‐growing provinces of the Philippines and is generally rare across those in China, with only limited exceedances in some southern and central Chinese provinces under SSP5‐8.5 by mid‐century.

Importantly, the projected increase in days with Tmax ≥ 37°C coincides with rice reproductive stages, when temperatures above 35°C can severely compromise pollen viability, spikelet fertility, and grain filling. Thus, the spatial and temporal intensification of extreme heat shown in Figures 1 and 2 reflects biologically consequential thresholds directly associated with yield instability and grain quality loss. Integrating climate projections with genomics‐enabled breeding therefore provides a compelling framework for accelerating the development of heat‐resilient rice cultivars.

## 3 EFFECTS OF HIGH TEMPERATURE ON RICE GROWTH AND DEVELOPMENT

Rice exhibits optimal growth and development within a temperature range of 25°C–28°C which supports stable cellular homeostasis and coordinated developmental gene expression (J. Yu et al., 2024). Heat stress responses in rice are highly stage specific, with experimentally defined thresholds reported across developmental phases. During anthesis, exposure to temperatures ≥35°C induces spikelet sterility through impaired pollen viability and anther dehiscence (Prasad et al., 2006). Similarly, Jagadish et al. (2007) demonstrated that short‐term exposure to 38°C during flowering significantly reduces spikelet fertility. At the booting stage, elevated temperatures disrupt microsporogenesis and pollen development, thereby reducing fertility, although specific threshold values vary among studies (Jagadish et al., 2008). During grain filling, high‐temperature stress alters starch biosynthesis and increases grain chalkiness, as demonstrated by physiological and transcriptomic analyses (Folsom et al., 2014; Wada et al., 2015). Importantly, heat stress effects depend not only on temperature intensity but also on stress duration: short‐term exposure primarily disrupts reproductive processes, whereas prolonged exposure causes cumulative physiological and metabolic damage. In addition, substantial genotypic variation in thermotolerance has been documented across rice germplasm, reflecting the diverse evolutionary and ecological origins of major subspecies. Traditional aus germplasm, including the widely studied donor N22, exhibits strong reproductive‐stage heat tolerance through the maintenance of spikelet fertility under stress (Jagadish et al., 2007). Indica varieties also display considerable variation in heat tolerance, often associated with adaptation to tropical environments, whereas japonica cultivars, which are typically adapted to temperate regions, tend to be more sensitive to elevated temperatures, particularly during flowering and grain filling (L. Zhao et al., 2016). These differences underscore the importance of exploiting diverse genetic resources to improve thermotolerance in rice breeding programs.

Prolonged exposure to elevated temperatures during early developmental stages impairs germination and seedling vigor. These effects are largely attributable to heat‐induced oxidative stress, which suppresses activities of key antioxidant enzymes, including superoxide dismutase (SOD), catalase (CAT), and peroxidase (POD) (Yin et al., 2008). Concurrently, high temperatures reduce expression of heat‐responsive genes such as *OsHSP18.6*, while genes primarily associated with other abiotic stresses such as *OsLEA3‐1* and *OsSOS1* remain minimally expressed, underscoring the specificity of stress‐dependent regulatory pathways (W. Hu et al., 2009).

During the seedling stage, sustained exposure to high temperature (38°C–45°C) results in excessive transpirational water loss, leaf yellowing, wilting, and compromised root development (J. Liu, Sun, et al., 2018). These phenotypes are often associated with the downregulation of *OsPIN1* and *OsARF4*, which affects auxin transport essential for proper root architecture (W. Li et al., 2022). Heat stress also inhibits ribulose‐1,5‐bisphosphate carboxylase/oxygenase (Rubisco) activase, thereby constraining photosynthetic activity. At the tillering stage, heat stress induces leaf curling, chlorosis, reduction in tiller numbers, and biomass accumulation (J. Xu, Henry, et al., 2020). These physiological impairments are associated with altered expression of *OsHSP70*, *OsHsfA2e*, *OsDREB2A*, as well as the enzymes involved in carbohydrate metabolism, including *OsSUS6*, *OsAGL2*, and *OsSSIVa*. Collectively, these changes disrupt sucrose cleavage, starch biosynthesis, and assimilate allocation to developing tillers.

High temperature during panicle initiation and microsporogenesis reduces spikelet number, size, and with spikelet sterility reaching up to 80% under exposure to temperatures ≥35°C–38°C for 3–5 days during anthesis (Jagadish, Muthurajan, Rang, et al., 2010; J. Xu, Henry, et al., 2020). The meiosis of pollen mother cells is particularly vulnerable as heat stress disrupts tapetum development by affecting genes/protein complexes such as *Tapetum Degeneration Retardation*, *EAT1*, *UDT1*, *OsMS1*, and *PTC1*. Disruption of these pathways leads to premature tapetal degeneration and nutrient starvation in microspores and subsequent pollen abortion (G. Liu, Zha, et al., 2020). During microsporogenesis and megasporogenesis, heat stress suppresses the expressions of protective molecular regulators such as *OsHSP101*, *OsHSP24.1*, members of the *MADS‐box* family (*OsMADS3*, *OsMADS58*), and bHLH (*OsbHLH148*, *OsbHLH116*) transcription factor (TF) families (Arshad et al., 2017). These perturbations compromise gametophyte viability, disrupt anthesis, pollination, and fertilization processes. Additionally, impaired pollen‐specific heat shock proteins (HSPs) reduce pollen count and viability, and altered expression of *OsEXPA4*/*OsEXPB2* inhibits pollen tube growth, resulting in reduced fertilization efficiency (F. Wang, Liu, et al., 2020).

Heat stress reduces stigma receptivity and disproportionately affects upper spikelets, reflecting spatial heterogeneity in heat‑responsive gene expression across the panicle. High temperature also interferes with endosperm development, resulting in reduced grain size and overall yield (J. Xu, Henry, et al., 2020). High temperatures during grain filling stage disrupt starch biosynthesis pathway, diminishing the activities of key enzymes such as AGPase, GBSSI, SBE, and starch synthase IIa (Folsom et al., 2014). These biochemical alterations lower amylose content from ∼19.8% under optimal conditions to ∼16.1% under heat stress (typically ≥30°C–35°C during grain filling over several days) and produce loosely packed starch granules. These changes are strongly associated with increased grain chalkiness, reduced milling quality, and yield penalties, as moderate warming (1°C–3°C) during grain filling has been reported to reduce yield by approximately 15%–21% (Folsom et al., 2014; Wada et al., 2015; C. Zhao et al., 2017). A summary of stage‐specific heat stress thresholds, exposure durations, and associated physiological and yield impacts in rice is provided in Table 1.

**TABLE 1:** Stage‐specific heat stress thresholds, exposure durations, and associated physiological and yield impacts in rice.

Development stage	Temperature threshold	Duration	Impact
Anthesis	≥35°C–38°C	3–5 days	50%–80% spikelet sterility
Microsporogenesis	≥35°C	Several days	Reduced pollen viability
Grain filling	≥30°C–35°C	Several days	Reduced amylose content, increased chalkiness
Grain filling	+1°C–3°C warming	Chronic	∼15%–21% yield reduction

While male reproductive processes such as pollen viability and anther dehiscence are widely recognized as primary determinants of heat‐induced sterility, increasing evidence indicates that female reproductive tissues and post‐fertilization processes are also highly vulnerable to elevated temperatures. Heat stress adversely affects stigma receptivity by reducing the secretion of extracellular matrix components and impairing pollen adhesion and hydration, thereby limiting successful pollen–pistil interactions (Hedhly, 2011; Jagadish et al., 2007). In addition, elevated temperatures disrupt ovule development and embryo sac formation, leading to abnormalities in mega gametogenesis and reduced fertilization efficiency (De Storme & Geelen, 2014; Snider & Oosterhuis, 2011).

Heat stress also alters the coordination between pollen tube growth and guidance within the pistil. High temperatures can impair pollen tube elongation, disrupt cytoskeletal organization, and interfere with signaling between male and female tissues, ultimately reducing fertilization success (Hedhly et al., 2009; Müller & Rieu, 2016). Furthermore, early seed development is particularly sensitive to thermal stress, where disruptions in endosperm cellularization, starch accumulation, and hormonal balance can lead to embryo abortion and reduced grain set (Folsom et al., 2014; Lafarge et al., 2017).

Collectively, these findings indicate that heat stress impacts both male and female reproductive functions as well as post‐fertilization development and that yield loss under high‐temperature conditions results from the combined failure of coordinated reproductive processes rather than male sterility alone.

### 3.1 Distinct effects of high night temperature

In addition to daytime heat stress, high night temperature (HNT) has emerged as an important constraint on rice productivity and grain quality. While daytime heat primarily impairs photosynthesis, pollen viability, and reproductive development, HNT exerts its effects predominantly through disruption of respiratory metabolism, carbon balance, and source‐sink dynamics. Elevated night temperatures significantly increase maintenance respiration rates, resulting in accelerated depletion of nonstructural carbohydrates accumulated during the day. This excessive respiratory carbon loss reduces net carbon gain, limits biomass accumulation, and ultimately leads to yield penalties even in the absence of severe daytime heat stress (Mohammed & Tarpley, 2009; Peng et al., 2004).

At the whole‐plant level, HNT alters the balance between carbon assimilation and utilization by decoupling daytime photosynthetic carbon fixation from nighttime carbon conservation. This imbalance weakens source–sink relationships, reducing assimilate availability for developing panicles and grains. Consequently, grain filling is compromised, leading to reductions in grain weight and harvest index. In addition, HNT has pronounced effects on grain quality through its impact on starch biosynthesis and deposition. Elevated night‐time respiration and associated metabolic shifts reduce the activity of key starch‐synthesizing enzymes, resulting in decreased amylose content, loosely packed starch granules, and increased grain chalkiness traits that directly affect milling quality and market value (Fitzgerald et al., 2009). At the cellular and biochemical levels, HNT has been linked to increased mitochondrial activity, altered ATP turnover, and enhanced oxidative stress due to sustained respiration, which may further disrupt metabolic homeostasis (Mohammed & Tarpley, 2009). Unlike acute daytime heat stress, which triggers rapid stress signaling responses, HNT represents a chronic metabolic stress that operates over extended periods, often without visible damage but with significant cumulative effects on yield and quality.

At the molecular level, emerging transcriptomic and physiological studies indicate that HNT responses involve partially distinct regulatory pathways compared to daytime heat stress. These include differential regulation of genes associated with respiration, carbohydrate metabolism, circadian clock components, and night‐time energy homeostasis, including *SnRK1*, α‐amylase genes (*Amy1A/Amy3D*), HSPs, and circadian‐associated regulators such as *OsPRR37* and *OsGI*, which have been implicated in carbon remobilization, respiratory adjustment, and temporal coordination of metabolic activity under elevated night temperatures (Jagadish et al., 2016). In particular, the interaction between temperature signaling and circadian regulation is increasingly recognized as a key determinant of HNT sensitivity, as elevated night temperatures can disrupt the temporal coordination of metabolic processes.

These findings demonstrate that HNT is not merely an extension of daytime heat stress, but rather a distinct physiological and molecular challenge. Accordingly, explicit differentiation between daytime and nighttime heat stress is essential for accurately characterizing thermotolerance mechanisms and for designing targeted breeding strategies to improve rice resilience under future climate scenarios, in which asymmetric warming, that is, disproportionately greater increases in nighttime temperatures, is becoming increasingly prevalent.

## 4 PHYSIOLOGICAL EFFECTS OF HIGH TEMPERATURE

### 4.1 Membrane damage

Exposure to high temperatures triggers excessive production of reactive oxygen species (ROS) in rice plants, leading to cellular damage. Elevated ROS levels compromise membrane integrity by increasing permeability and disrupting structural stability (Ishimaru et al., 2019). Heat stress also alters membrane lipid composition, increasing the proportion of saturated fatty acids while reducing unsaturated fractions, thereby decreasing membrane fluidity and promoting protein denaturation (Bita & Gerats, 2013; Higashi & Saito, 2019). Additionally, heat stress suppresses fatty acid desaturase activity, further limiting lipid unsaturation and accelerating membrane destabilization (Niu & Xiang, 2018).

**FIGURE 4:**
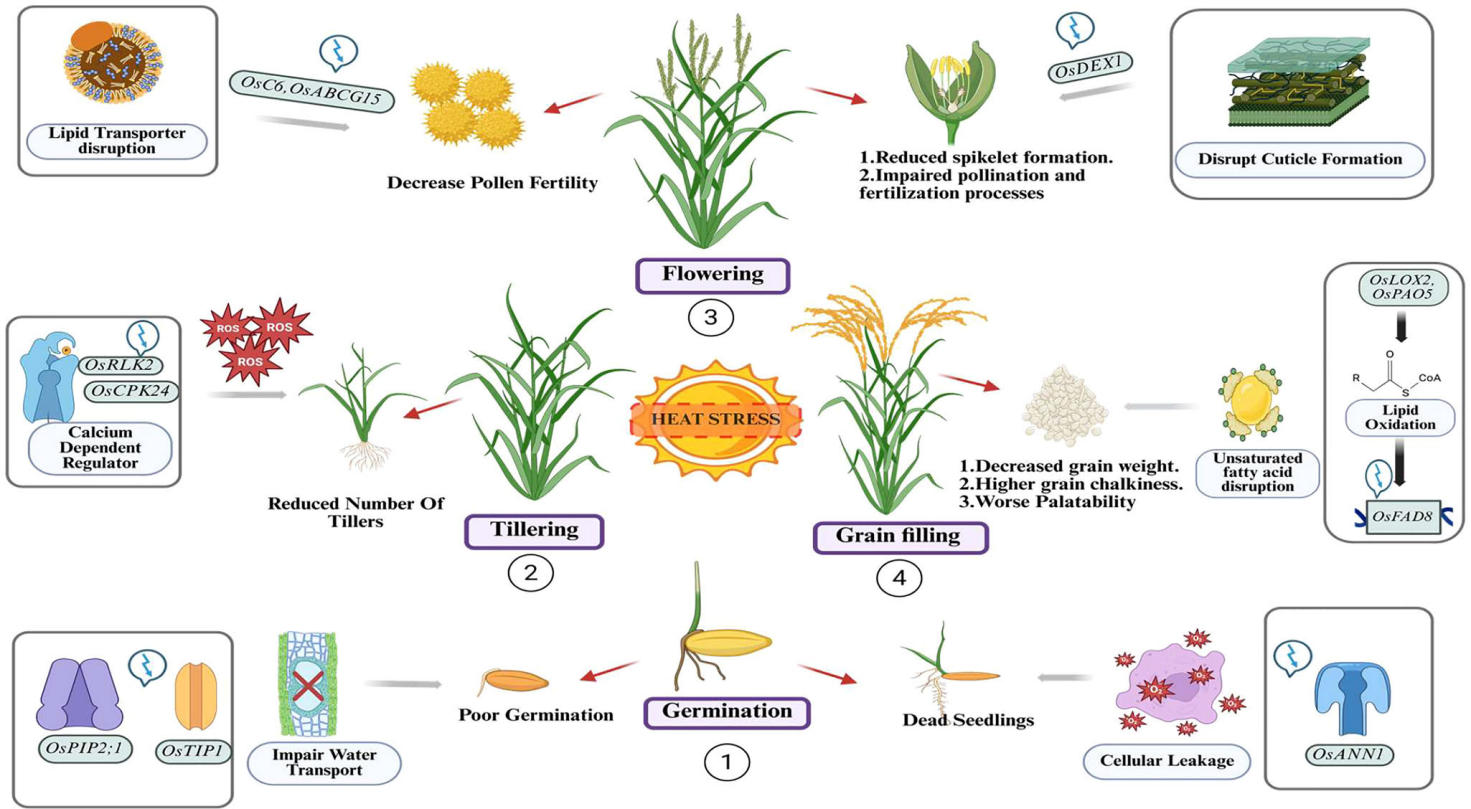
Stage‐specific molecular and physiological impacts of heat stress across four key developmental stages of rice. This figure illustrates the sequential progression from germination to grain filling while emphasizing the cumulative and interconnected effects of high temperature across stages. (1) *Germination*: Heat stress downregulates the aquaporin genes *OsPIP2;1* and *OsTIP1*, impairing water uptake and germination. Concurrent suppression of *OsANN1*, which contributes to membrane stability and antioxidant defense, increases cellular leakage and reduces seedling viability. (2) *Tillering*: Elevated temperatures drive the accumulation of reactive oxygen species (ROS), which disrupt calcium‐dependent signaling mediated by *OsRLK2* and *OsCPK24*. The resulting impairment of signal transduction limits tiller formation and vegetative growth. (3) *Flowering*: Reproductive development is severely compromised. Disruption of the lipid transporter genes *OsC6* and *OsABCG15* impairs anther cuticle formation, while *OsDEX1*, required for pollen exine development, is also affected. Together, these defects reduce pollen viability, hinder pollination, and increase spikelet sterility. (4) *Grain filling*: Prolonged heat stress perturbs lipid metabolism and membrane integrity. Upregulation of the lipid‐oxidation genes *OsLOX2* and *OsPAO5* accelerates membrane degradation, while downregulation of *OsFAD8* reduces unsaturated fatty acid synthesis and compromises membrane fluidity. The combined effect lowers grain weight, increases chalkiness, and degrades grain quality and palatability.

Biochemical changes under heat stress in rice manifest as distinct phenotypic injuries across developmental stages (Figure 4). During early germination, reduced expression of aquaporin genes, *OsPIP2;1* and *OsTIP1;1*, impairs water transport (Sakurai et al., 2005), while insufficient accumulation of the membrane‐repair protein *OsANN1* increases susceptibility to cellular leakage and oxidative damage (Qiao et al., 2015). At the tillering stage, persistent accumulation of ROS diminishes the expression of calcium‐dependent regulators *OsCPK24* and *OsRLK2*, resulting in fewer tillers and reduced vegetative biomass (Y. Liu, Xu, et al., 2018).

The most severe effects occur during flowering, where downregulation of pollen lipid‐transport genes *OsC6*, *OsABCG15*, and *OsDEX1* disrupts anther cuticle formation, leading to pollen sterility and spikelet abortion (L. Wu et al., 2014). In the grain‐filling stage, intensified lipid oxidation, driven by *OsLOX2* and *OsPAO5*, causes membrane peroxidation (J. Huang et al., 2014), while reduced expression of *OsFAD8* limits unsaturated fatty acid pools, resulting in chalky, poorly filled grains (Gopalakrishnan Nair et al., 2009). Conversely, genotypes with elevated expression of heat‐shock proteins HSP101, HSP90, and HSP70 (Lin et al., 2014) and protein quality‐control E3 ligases *heat‐induced RING finger protein 1* (*OsHIRP1*), *heat and cold induced 1* (*OsHCI1*), and *OsTT1* maintain membrane stability and exhibit greater heat tolerance with improved grain quality (Lim et al., 2013).

### 4.2 Photosynthetic damage

Elevated temperatures significantly disrupt the photosynthetic machinery in rice, with photosystem II (PSII) being especially vulnerable to thermal stress. Exposure to heat increases the permeability of thylakoid membranes, leading to a reduction in chlorophyll concentration (Chaudhary et al., 2021). This change causes grana to become unstacked, pigments to be lost, and light‐harvesting efficiency to decline markedly (X. Wang et al., 2017). The photochemical phase of photosynthesis is severely affected, as evidenced by a decrease in the variable to maximum fluorescence ratio (Fv/Fm), a key indicator of PSII performance. As a result, the overall rate of photosynthesis drops sharply under sustained heat exposure (Q. Wang, Chen, et al., 2018).

Further, heat stress induces oxidative damage that impairs the oxygen‐evolving complex (OEC), thereby inhibiting electron donation to the PSII reaction centers and restricting downstream electron transport. This leads to reduced energy conversion efficiency (Sailaja et al., 2015). In addition, elevated temperatures suppress the carbon fixation process by decreasing the activity of Rubisco, and this suppression is primarily attributed to the thermal inactivation of Rubisco activase, the enzyme responsible for maintaining Rubisco in its catalytically active form (Ogbaga et al., 2018). Under heat stress, Rubisco activase loses its conformational stability, exhibits diminished ATP‐dependent turnover, and fails to effectively carbamylate Rubisco, thereby limiting carboxylation efficiency and CO_2_ assimilation (Chaudhary et al., 2021; Perdomo et al., 2017). Collectively, these disruptions, PSII damage, OEC instability, and the deactivation of Rubisco/Rubisco activase drive the collapse of photosynthesis under high‐temperature stress.

### 4.3 Overproduction of ROS under heat stress

Heat stress disrupts cellular redox homeostasis and leads to the accumulation of ROS, which acts as crucial messengers to activate stress‐response pathways in plants. In rice, exposure to elevated temperatures during meiosis increases ROS levels in anthers by up to threefold compared to optimal conditions (Q. Zhao, Zhou, et al., 2018). While moderate levels of ROS support adaptive signaling, excessive ROS overwhelms cellular defenses and causes oxidative injury. To counteract this oxidative stress, plants employ complementary antioxidative systems that include both enzymatic and nonenzymatic detoxifiers. Nonenzymatic ROS scavengers such as carotenoids, ascorbate, and glutathione play vital roles: carotenoids quench singlet oxygen and dissipate excess excitation energy; ascorbate directly reduces harmful ROS intermediates in the water–water cycle; and glutathione participates in redox buffering and regeneration of oxidized antioxidants (P. Rana et al., 2024). In parallel, enzymatic detoxification is mediated by SOD, which catalyzes the conversion of superoxide radicals (O_2_^−^) into hydrogen peroxide (H_2_O_2_). CAT then rapidly converts H_2_O_2_ into water and oxygen, preventing the formation of cellular hydroxyl radicals. Ascorbate peroxidase (APX) further detoxifies H_2_O_2_ using ascorbate as an electron donor, while monodehydroascorbate reductase regenerates reduced ascorbate from its oxidized form, thereby sustaining the ascorbate–glutathione cycle and maintaining redox homeostasis (Gill & Tuteja, 2010).

Heat stress induces stress‐responsive transcriptional factors in rice pistils, most notably respiratory burst oxidase homologs (RBOHs). These enzymes function upstream in the signaling pathway, generating ROS that trigger controlled oxidative signals essential for plant acclimation (C. Zhang, Li, et al., 2018). Under elevated temperatures, RBOHs produce localized ROS pulses that activate downstream defense genes, initiate Ca^2^^+^‐dependent signal amplification, and regulate redox‐linked transcription networks. As a result, they serve as early signaling hubs rather than direct scavengers.

Within this defense hierarchy*, OsANN1* operates downstream, contributing to cellular protection by modulating antioxidant output (F. Zhang et al., 2021). Upregulation of *OsANN1* during heat stress is associated with increased activity of SOD and CAT, enhanced stabilization of membrane integrity, and reduced accumulation of toxic ROS species (Qiao et al., 2015). This indicates that *OsANN1* not only promotes the activity of scavenging enzymes but may also act as a calcium‐binding regulator, influencing ROS‐responsive pathways and helping to prevent oxidative collapse during prolonged heat stress.

When ROS production exceeds the cell's detoxification capacity, oxidative stress intensifies, resulting in lipid membrane disruption, protein denaturation, and ultimately programmed cell death (Niu & Xiang, 2018). Excess ROS accelerates lipid peroxidation and protein oxidation, increasing accumulation of malondialdehyde (MDA), a key biochemical marker of oxidative injury (Chakraborty & Bhattacharjee, 2015). Elevated MDA further disrupts nucleic acid structure and enzyme stability, aggravating cellular deterioration. Together, ROS levels and MDA content serve as reliable indicators of oxidative severity and membrane damage under heat stress (Sailaja et al., 2015).

Importantly, the role of ROS under heat stress is highly context dependent, as ROS function both as essential signaling molecules and as mediators of cellular damage. Under moderate or transient heat stress, ROS serve as key secondary messengers that activate Ca^2^^+^ signaling, heat shock transcription factors (HSFs), and downstream defense pathways, thereby promoting acclimation responses (Mittler et al., 2011; Suzuki et al., 2012). This signaling function is tightly regulated in both space and time, allowing controlled ROS bursts to initiate protective mechanisms without causing cellular injury. However, under prolonged or severe heat stress, ROS accumulation exceeds the scavenging capacity of antioxidant systems such as SOD, CAT, and APX, resulting in oxidative damage to lipids, proteins, and nucleic acids (Gill & Tuteja, 2010). The transition from beneficial signaling to cytotoxicity is therefore governed by the balance between ROS production (e.g., via RBOHs) and detoxification capacity, as well as by the intensity and duration of heat exposure. This dual role underscores the importance of redox homeostasis, in which precise regulation of ROS levels determines whether cells achieve thermotolerance or undergo oxidative stress‐induced damage.

### 4.4 Disruption of carbohydrate metabolism

Heat stress disrupts carbohydrate metabolism and alters the distribution of photo‐assimilates within plants, resulting in energy deficits and impaired reproductive development (Gammulla et al., 2010). Glycolysis is suppressed due to the downregulation of key metabolic enzymes, such as phosphofructokinase and phosphoglucose isomerase, both of which are essential for ATP production and cellular energy balance (X. Li, Lawas, et al., 2015). Under heat stress, rice anthers exhibit reduced sugar content, limiting the nutritional supply necessary for pollen development and maturation, resulting in reduced pollen viability and failure of fertilization (Rezaul et al., 2019). Notably, the expression of *carbon starved anther* is higher in heat‐susceptible rice varieties, whereas the sugar transporter *OsMST8* shows increased expression in heat‐tolerant varieties, helping to maintain robust sugar transport and metabolism (X. Li, Lawas, et al., 2015).

Heat stress disrupts carbohydrate metabolism in rice by suppressing cell wall invertase activity, primarily regulated by *GIF1*/*OsCIN2*, along with isoforms such as *OsCIN1* and *OsCIN4*. Reduced *GIF1*/*OsCIN2* activity limits sucrose cleavage into glucose and fructose, decreasing soluble sugars required for grain filling. Heat stress also impairs sucrose synthase (SuSy), particularly *OsSUS2*, *OsSUS3*, and *OsSUS4*, reducing UDP‐glucose production for starch biosynthesis (Russel French et al., 2014). Heat‐sensitive genotypes show altered levels of sucrose and raffinose‐family oligosaccharides, whereas tolerant cultivars maintain higher nonstructural carbohydrate levels and stronger *GIF1/OsCIN2* and *OsSUS* activity, supporting efficient carbon allocation and grain filling under high temperature (Bahuguna et al., 2017).

### 4.5 Phytohormone imbalance

Heat stress disrupts phytohormone homeostasis, and this hormonal imbalance is one of the most critical factors influencing reproductive success in rice under elevated temperatures (C. Wu et al., 2016). Among all plant hormones, cytokinins (CTKs), auxin, and abscisic acid (ABA) are the major regulators affected during heat stress. Under these conditions, spikelets exhibit reduced CTK levels due to the downregulation of IPT (isopentenyl transferase) biosynthetic genes and the concomitant up‐regulation of CTK oxidase/dehydrogenase genes (CKX family), which accelerate CTK degradation (C. Wu et al., 2016, 2017). This suppression limits meristem activity, spikelet initiation, and cell proliferation, ultimately reducing panicle size and increasing the risk of spikelet sterility. IAA concentrations decline significantly in developing florets because heat stress interferes with auxin biosynthesis genes (YUCCA family) and transporters such as *OsPIN1* and *OsPIN5c*, resulting in impaired pollen germination, reduced anther dehiscence, and weaker kernel filling. However, cultivars that maintain stable expression of IPT and YUCCA genes or sustain PIN‐mediated polar transport demonstrate improved spikelet fertility under high temperatures (Sakata et al., 2010). Conversely, ABA levels rise sharply during heat stress, driven by increased transcription of ABA biosynthetic genes such as *OsNCED3* and *OsNCED5*, particularly in anthers and developing grains (M. Li, Li, et al., 2018). Excess ABA suppresses pollen tube growth, accelerates tapetal degeneration, and reduces seed set, positioning ABA as a central hormonal trigger for heat‐induced sterility. Brassinosteroids (BRs) and salicylic acid (SA) can partially counteract these effects by activating antioxidant pathways and stabilizing reproductive development, but the dominance of ABA under heat stress often overrides these recovery mechanisms.

## 5 MECHANISMS OF PLANT RESPONSE TO HEAT STRESS

Plants respond to heat stress by remodeling their transcriptional, proteomic, and metabolic pathways to maintain cellular homeostasis and metabolic function (Poór et al., 2022). Under heat stress, approximately 5% of the plant transcriptome is strongly induced, with upregulated transcripts encoding factors involved in metabolic regulation, translation, gene expression, and signal transduction, including hormonal, calcium, and lipid signaling cascades (Scharf et al., 2012). Central to thermotolerance are the HSFs, which act as master regulators by activating protective pathways and multiple defense‐associated genes (Mishra et al., 2002). Of the three major HSF classes—HSFA, HSFB, and HSFC‐ the HSFA subclass serves as the primary activator. Members of the HSFA1 (such as HSFA1a, HSFA1b, HSFA1c, HSFA1d) function as frontline regulators, rapidly initiating transcriptional activation in response to heat. Downstream, HSFA2, HSFA3, and HSFA6b contribute to sustained or long‐term acquired thermotolerance, while HSFB1 and HSFB2 mainly act as co‐regulators or repressors, fine‐tuning the heat response.

In addition to TFs, heat stress triggers the rapid accumulation of HSPs, key molecular chaperones involved in protein stabilization and refolding. These include HSP100, HSP90, HSP70, HSP60, HSP40, and small heat shock proteins (sHSPs), all of which help prevent protein misfolding, aggregation, and proteotoxicity (W. Wang et al., 2004). Within the HSF family, HSFA1 members initiate the transcription of numerous heat‐responsive genes encoding these HSPs and other protective proteins (Mishra et al., 2002). However, chaperones represent only a subset of heat stress‐induced transcripts, indicating additional layers of thermotolerance regulation and suggesting the presence of yet‐undefined transcriptional control elements (Ohama et al., 2017).

### 5.1 Signaling cascades in response to heat stress

Plants sense heat stress through rapid changes in calcium ion (Ca^2^^+^) concentration, membrane fluidity, and redox status, which are then translated into transcriptional responses (Fortunato et al., 2023). Research in the heat‐sensitive moss *Physcomitrella patens* has shown that plants can sense small temperature shifts by activating specific Ca^2^^+^ channels, resulting in a rapid increase in cytosolic Ca^2^^+^ levels (Saidi et al., 2009). In higher plants, the cell wall acts as the initial structural barrier, but under heat stress, it undergoes remodeling that leads to the release of apoplastic Ca^2^^+^ and a subsequent rise in cytosolic Ca^2^^+^ (H. Wu et al., 2018). A key enzyme in this process is pectin methylesterase, which is upregulated during heat stress and promotes pectin demethylesterification. This acidifies the cell wall and enhances endopolygalacturonase activity, loosens the cell wall matrix, and transiently releases Ca^2^^+^ from Ca‐pectate complexes into the apoplast and then into the cytosol (H. C. Wu & Jinn, 2010). Simultaneously, heat‐induced changes in plasma membrane fluidity activate Ca^2^^+^‐permeable channels, particularly cyclic nucleotide‐gated channels (CNGC) *OsCNGC14 and OsCNGC16* in rice, which mediate heat‐triggered Ca^2^^+^ influx and are essential for full thermotolerance (Baxter et al., 2014; Clough et al., 2000). These CNGCs possess multiple transmembrane segments, a cytosolic cyclic nucleotide–binding domain, and a conserved calmodulin‐binding region, enabling them to integrate both cyclic nucleotide and Ca^2^^+^/calmodulin (CaM) signals. The Ca^2^^+^ signal is then decoded by calmodulin and CaM‐like proteins, for example, *AtCaM3* in *Arabidopsis* (*Arabidopsis thaliana* (L.) *Heynh*) acts as a positive regulator of heat stress signaling, and its loss reduces thermotolerance, while overexpression enhances survival under heat (Clough et al., 2000; Saidi et al., 2009). In rice, Ca^2^^+^ signals are further relayed by Ca^2^^+^‐dependent protein kinases (CDPKs) and CaM‐dependent kinases, which phosphorylate downstream components, including HSFs such as *OsCDPK24*/*OsCDPK28* acting on *OsHSFA4d* (Y. Fang et al., 2025).

Heat‐induced changes in membrane fluidity initiate lipid signaling and ROS production (Baxter et al., 2014). At the plasma membrane, RBOHs, such as *AtRBOHD* in *Arabidopsis*, *OsRbohB/OsRbohH* in rice, function as NADPH oxidases, converting cytosolic electrons into apoplastic ROS, particularly H_2_O_2_. These act as primary signal generators rather than just downstream markers (Kimura et al., 2012). Structurally, RBOHs possess a cytosolic C‐terminal region with FAD and NADPH‐binding sites and an N‐terminal regulatory domain with EF‐hand Ca^2^^+^‐binding motifs and multiple phosphorylation sites, allowing for Ca^2^^+^/CaM and kinase‐mediated activation (Kimura et al., 2012). Upon heat perception, Ca^2^^+^ influx *via OsCNGC14/16* and Ca^2^^+^‐binding annexin *OsANN1* activates RBOHs, inducing a rapid ROS burst that propagates as ROS waves through tissues and into systemic leaves.

At the transcriptional level, the AP2/ERF TF ERF74 has been identified as a key upstream regulator of RBOH expression (Ma et al., 2024). ERF74 binds to GCC‐box motifs in the RBOHD promoter and acts as an on–off switch controlling RBOH‐dependent ROS burst during stress. Loss‐of‐function mutations in ERF74 (or combined ERF74/ERF75 mutants) significantly reduce heat‐induced RBOH expression and ROS production (Devireddy et al., 2021). ERF74 also enhances the transcription of ROS‐detoxifying genes during later heat stress phases in an RBOH‐dependent manner positioning the ERF74–RBOH–ROS module as a central regulator of both early ROS bursts and downstream antioxidant gene activation (Mittler et al., 2011).

Downstream of Ca^2^^+^/ROS signaling, HSFs and HSPs form the core of the heat‐response network (Kotak et al., 2007). Under nonstress conditions, key activator HSFs (such as *HSFA1s*) are kept inactive through association with HSP70/HSP90 chaperone complexes. Upon heat stress, rapid protein unfolding and changes in redox state titrate HSP70/HSP90 away from HSFA1, allowing HSFs to trimerize, translocate into the nucleus, and bind heat shock elements (HSEs) in promoters of target genes. This leads to strong induction of HSP101, HSP90, HSP70, and various sHSPs, which refold denatured proteins and stabilize membranes and organelles. Regulated ROS accumulation, particularly H_2_O_2_ generated by RBOHs, further contributes to HSF activation; several HSFs can act as direct H_2_O_2_ sensors, where oxidative modification promotes HSF trimer assembly and DNA binding, thereby linking ROS signaling to transcriptional control of heat‐responsive genes (L. Wang et al., 2014). Finally, ROS levels are tightly balanced by enzymatic antioxidants such as ascorbate POD, CAT, and SOD, many of which are themselves transcriptionally regulated by HSFs and ROS‐activated TFs (Kimura et al., 2012; L. Wang et al., 2014). Together, *OsCNGC14/16*–Ca^2^^+^/CaM–CDPKs–RBOH–ERF74–HSF–HSP–APX/CAT/SOD form a sequential signaling cascade in which Ca^2^^+^ and ROS act upstream messengers, HSFs and HSPs constitute the central execution module, and antioxidant enzymes and chaperones collectively determine whether cells achieve thermotolerance or succumb to heat‐induced damage.

Although several components of heat stress signaling and transcriptional regulation have been extensively characterized in *A. thaliana*, their functional conservation in rice varies among pathways. Core regulatory modules, including Ca^2^^+^ signaling, ROS‐mediated signaling, and HSF–HSP networks, are well supported in rice. In contrast, other mechanisms, particularly transcriptional interactions and epigenetic regulation, are frequently inferred from *Arabidopsis* studies and remain insufficiently validated in rice. Caution is therefore warranted when extrapolating these mechanisms across species.

### 5.2 Lipid signaling

Lipid‐mediated signaling plays a central role in plant adaptation to heat stress and is often initiated by temperature‐induced changes in plasma membrane fluidity (Sharma et al., 2023). Elevated temperatures activate lipid‐modifying enzymes like phospholipase D (PLD) and phosphatidylinositol‐4‐phosphate 5‐kinase (PIPK), which drives the production of Ca^2^^+^‐dependent bioactive lipid messengers. Heat stress triggers the mobilization of internal Ca^2^^+^ stores, further amplifying the signaling cascade. As a result, lipid‐derived compounds such as phosphatidic acid, phosphatidylinositol‐4,5‐bisphosphate (PIP_2_), and inositol trisphosphate (IP_3_) accumulate and promote plant's thermal resilience (Mishkind et al., 2009). Genetic evidence shows that a reduction in IP_3_ concentration diminishes thermotolerance by decreasing the activity of sHSPs. Consistently, knockout mutations in phospholipase C9 (PLC9) and PLC3‐enzymes required for IP_3_ biosynthesis markedly reduce heat‐induced cytosolic Ca^2^^+^ influx, resulting in impaired thermotolerance. Furthermore, IP_3_ can be phosphorylated into inositol hexakisphosphate (IP_6_), which promotes Ca^2^^+^ release from intracellular stores. However, the precise mechanism by which Ca^2^^+^ influx regulates lipid signaling pathways under heat stress remains unclear (H. C. Wu & Jinn, 2012).

### 5.3 Transcriptional control of heat stress response

**FIGURE 5:**
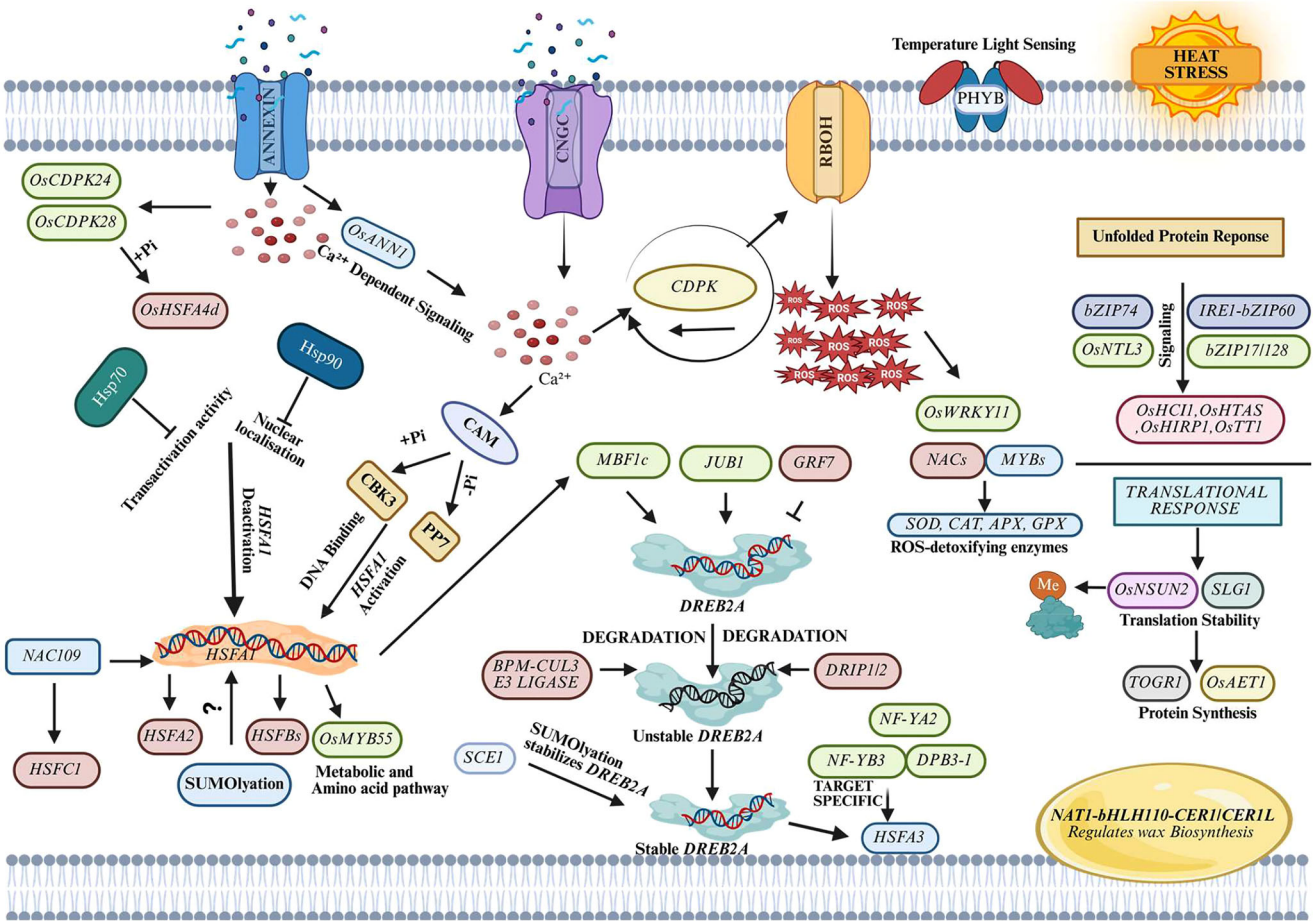
Overview of the signaling pathways and transcriptional regulatory networks mediating heat stress tolerance in rice. (1) *Heat perception*: Elevated temperatures alter plasma membrane fluidity, activating annexin (*OsANN1*) and cyclic nucleotide‐gated channels (CNGCs), resulting in rapid Ca^2^^+^ influx and initial reactive oxygen species (ROS) production via respiratory Burst Oxidase Homologs (RBOHs). (2) *Signal cascading*: Ca^2^^+^‐ROS crosstalk activates calcium‐dependent protein kinases (*OsCDPK24/28*), which phosphorylate transcriptional regulators such as OsHSFA4d. (3) *Transcriptional activation*: Master regulators *HSFA1* are released from HSP70/HSP90 complexes and induce downstream transcription factors (HSFA2, HSFBs, DREB2A, NAC, MYB, WRKY), forming a hierarchical regulatory network. (4) *Protein and redox homeostasis*: Heat shock proteins (HSPs), antioxidant enzymes (superoxide dismutase [SOD], catalase [CAT], ascorbate peroxidase [APX]), and metabolic protectants are upregulated to maintain proteostasis and redox balance. (5) *Post‐transcriptional and post‐translational regulation*: DREB2A activity is fine‐tuned by SUMOylation and ubiquitin‐mediated degradation (DRIP1/2, BPM–CUL3), ensuring precise stress response control. (6) *Unfolded protein response (UPR)*: Endoplasmic reticulum (ER) stress activates IRE1–bZIP60, bZIP17/28, and *OsNTL3* pathways, inducing ER chaperones (*OsHCI1*, *OsHTAS*, *OsHIRP1*, *OsTT1*). (7) *Translational regulation and structural adaptation*: RNA methylation (*OsNSUN2*), ribosome stability (TOGR1), and wax biosynthesis (*NAT1–bHLH110–CER1/CER1L*) contribute to sustained thermotolerance.

Plants rely on a sophisticated transcriptional network to manage heat stress, with HSFs playing a central role. These factors activate a broad array of defense‐related genes, including other TFs, antioxidant enzymes, and HSPs, all of which are essential for thermotolerance. In rice, a total of 25 HSFs has been identified and grouped into three major classes, namely HSFA, HSFB, and HSFC (Mittal et al., 2009). Notably, 22 of these genes are significantly upregulated in response to elevated temperatures, highlighting their active role in heat adaptation. Among these, the HSFA1 subfamily serves as the primary regulatory node at the top of the transcriptional hierarchy, initiating the heat stress response by promoting the expression of downstream genes (Figure 5). In *A. thaliana*, combined mutations in *AtHSFA1a*, *AtHSFA1b*, and *AtHSFA1d* drastically impair heat‐responsive gene activation, demonstrating that HSFA1s function as master regulators of thermotolerance (T. Yoshida et al., 2011). In rice, several HSF genes, including *OsHsfA2a*, *OsHsfA2d*, and *OsHsfA2e*, are strongly induced under heat stress and are proposed to regulate thermotolerance (Mittal et al., 2009; Yokotani et al., 2008). Other key regulators include HSFA2, various HSFBs, and proteins such as DREB2A (dehydration‐responsive element binding protein 2A) and MBF1c (multiprotein bridging factor 1C), which further enhance heat resilience. Alternative splicing also contributes to thermotolerance. In rice, *OsHSFA2d* undergoes heat‐induced splicing to form an active variant that stimulates HSP gene expression (Y. Fang et al., 2025). Similarly, transgenic *Arabidopsis* overexpressing *OsHSFA2e* shows increased HSP production and improved heat tolerance, underscoring the conserved nature of HSF functions (Yokotani et al., 2008). *OsHSFB2b* is another gene strongly induced by heat, indicating its regulatory significance.

Beyond the HSF family, TFs from other families integrate into the heat response network. The ERF/AP2 family member *DREB2A* is directly controlled by *HSFA1* and participates in forming transcriptional complexes (Rehman & Mahmood, 2015). In *Arabidopsis*, DREB2A collaborates with NF‐YA2, NF‐YB3, and DPB3‐1 to activate HSFA3 expression by binding to its promoter, and analogous regulatory interactions have been suggested in rice, although direct validation remains partial (Yokotani et al., 2008). A similar mechanism operates in rice, where *OsDREB2B* partners with *OsDPB3‐2* and interacts with NF‐YA2 and NF‐YB3 to regulate *HSFA3*, reinforcing stress signaling pathways (Sato et al., 2016). *MBF1c* is another pivotal component with its expression directly regulated by *HSFA1b* through HSEs in its promoter region (D. Qin et al., 2015). *MBF1c* expression rises quickly during heat stress and modulates the activity of *DREB2A*, *HSFB2a*, and *HSFB2b* by targeting their promoters. Experimental evidence from yeast and rice indicates that *MBF1c* overexpression enhances thermotolerance, confirming its functional conservation across species (D. Qin et al., 2015). Recent studies have expanded this regulatory network further. *OsCDPK24* and *OsCDPK28*, two calcium‐dependent protein kinases, have been shown to phosphorylate OsHSFA4d, thereby modulating its activity and linking calcium signaling to transcriptional regulation under both abiotic and biotic stress conditions. This phosphorylation event fine‐tunes the HSF‐mediated transcriptional cascade, integrating heat response with broader stress signaling pathways in rice (Y. Fang et al., 2025).

Several key genes have been identified in rice that contribute to heat stress tolerance through diverse regulatory mechanisms (Table 2). For example, *QT12* acts as a negative regulator of thermotolerance; silencing this gene enhances yield under heat stress (W. Li et al., 2025). ERF74, ERF77, ERF108, and ERF125 are TFs that activate HsfA2c and other heat‐responsive genes, enhancing thermotolerance (Luo et al., 2025). *Os11g0244200*, *Os01g0135800*, and *Os04g0445100* are *HSP20/α‐crystallin* family genes involved in protein stabilization under heat stress (Nyasulu et al., 2025). The *NAT1‐bHLH110‐CER1/CER1L* module regulates wax biosynthesis for enhanced thermotolerance without yield penalties (H. Lu et al., 2025). Regulation of these TFs is not limited to gene expression alone; protein interactions and posttranslational modifications also play key roles. In *Arabidopsis*, AtHSP70 and AtHSP90 interact with AtHSFA1, restricting its nuclear localization and function. In rice, OsHSBP1 and OsHSBP2 act similarly by binding to activated HSFs and functioning as negative regulators (M. Guo et al., 2016). When overexpressed, these proteins diminish plant survival under heat stress, suggesting help in fine‐tuning the stress response (R. Rana et al., 2012). Posttranslational changes such as phosphorylation and SUMOylation further adjust TF activity. For instance*, OsHSFA1a* is activated through phosphorylation by *AtCBK3* in *Arabidopsis*, which enhances its DNA‐binding ability and similar regulatory mechanisms are hypothesized in rice but require further experimental validation (H.T. Liu et al., 2008). Conversely, SUMOylation inhibits *HSFA2*, decreasing its transcriptional activity (Cohen‐Peer et al., 2010). Overexpressing *OsSIZ1‐a*, a SUMO E3 ligase, increases heat tolerance in rice, *Arabidopsis*, and cotton (*Gossypium hirsutum L*.). SUMOylation also contributes to DREB2A stabilization, adding another layer of regulation to heat tolerance mechanisms (F. Wang, Liu, et al., 2020). NAC TFs are also vital in orchestrating the heat stress response (Fuertes‐Aguilar & Matilla, 2024). *OsNAC19* acts upstream by directly targeting HSF promoters. *JUNGBRUNNEN1* (*JUB1*), a well‐studied NAC gene in *Arabidopsis*, responds to oxidative stress signals and enhances *DREB2A* expression (Fuertes‐Aguilar & Matilla, 2024). Its rice counterpart, *ONAC066*, plays a similar role by activating *OsDREB2A* under stress conditions. Moreover, other key TFs, such as *OsMYB55*, *OsbZIP46*, *SNAC3*, and *OsWRKY11*, operate independently of HSFA1 and contribute to heat adaptation, reflecting the complexity and redundancy of the plant's transcriptional defense strategy (X. Wu et al., 2009).

**TABLE 2:** A curated list of major heat stress‐responsive genes in rice, including their functions.

Gene	ID/locus	Gene type	Function	Validation	Ref
*OsHsfA2e*	Os03g0795900	HSF TF	Activates HSP genes; enhances thermotolerance	OE	(Yokotani et al., 2008)
*OsWRKY11*	Os01g0626400	WRKY TF	Improves thermotolerance via ROS scavenging	OE	(X. Wu et al., 2009)
*OsHSBP1/2*	Os09g0375100, Os06g0274000	HSF‐binding proteins	Negative regulator of HSF activity	OE	(R. Rana et al., 2012)
*OsHIRP1*	Os03g0302200	RING E3 ubiquitin ligase	E3 ligase mediating protein degradation during heat stress	OE	(Lim et al., 2013)
*OsMYB55*	Os05g0553400	MYB TF	Regulates stress‐responsive metabolic genes	OE	(El‐Kereamy et al., 2012)
*OsTT1*	Os03g0387100	Proteasome subunit	26S proteasome subunit involved in protein clearance under heat	OE/INT	(X.‐M. Li, Chao, et al., 2015)
*OsANN1*	Os01g0497400	Annexin protein	Annexin protein modulating ROS scavenger enzymes	OE	(Qiao et al., 2015)
*OsNTL3*	Os01g0261200	NAC TF	Transcription factor activating OsbZIP74 under heat stress	OE	(X. H. Liu, Lyu, et al., 2020)
*OsHTAS*	Os09g0323100	RING E3 ubiquitin ligase	Regulates seedling survival under heat stress	OE	(J. Liu et al., 2016)
*OsHsfA2d*	Os04g0492900	HSF TF	Alternative splicing under heat triggers unfolded protein response (UPR)	OE	(Cheng et al., 2015)
*TaMBF1c*	TraesCS3D02G240200	Coactivator protein	Bridges HSF and DREB transcriptional pathways	OE (in rice)	(D. Qin et al., 2015)
*OsDREB2A*	Os01g0165000	DREB TF	Controls downstream heat‐responsive genes	OE	(F. Wang, Liu, et al., 2020)
*OsbZIP74*	Os06g0622700	bZIP TF	Key transcription factor mediating UPR under heat	OE	(S. Lu et al., 2012)
*OsNSUN2*	Os09g0471900	RNA methyltransferase	m^5^C RNA methyltransferase ensuring translation under heat	KO + Comp	(Y. Tang et al., 2020)
*TOGR1*	Os03g0669000	RNA helicase	Stabilizes rRNA and ribosome function under high temperature	KO + Comp	(D. Wang et al., 2016)
*OsSLG1*	Os12g0588900	tRNA thiolation enzyme	Confers seedling and reproductive stage thermotolerance	KO + Comp	(Y. Xu, Zhang, et al., 2020)
*NAT1–bHLH110–CER1/CER1L*	Os04g0512200	TF–enzyme module	Regulates wax biosynthesis and enhances heat stress tolerance via cuticular wax formation	KO (CRISPR) + OE (bHLH110)	(H. Lu et al., 2025)
*QT12*	Os12g0173366	QTL gene	Negative regulator of grain‐quality thermotolerance; silencing enhances yield under heat	KO (CRISPR) + Comp	(W. Li et al., 2025)
*ERF74*, *ERF77*, *ERF108*	Os05g0497300, Os04g0610400, Os01g0131600	ERF TF family	Transcription factors activating HsfA2c and other heat‐responsive genes	OE + DKO	(Luo et al., 2025)
*Hsp20*	Os11g0244200, Os01g0135800, Os04g0445100	Small HSPs	HSP20/α‐crystallin family genes involved in protein stabilization under heat stress	Expression‐based association (no OE/KO validation)	(Nyasulu et al., 2025)
*OsCDPK24*, *OsCDPK28*	Os11g0171500, OOs12g0169800	CDPK kinases	Calcium‐dependent protein kinases that phosphorylate OsHSFA4d to coordinate abiotic and biotic stress responses	SKO + DKO + OE	(Y. Fang et al., 2025)

Abbreviations: CDPK, Ca^2^^+^‐dependent protein kinase; Comp, complementation; CRISPR, Clustered Regularly Interspaced Short Palindromic Repeats; DKO, double knockout mutants; DREB, dehydration‐responsive element binding; HSF, heat shock transcription factor; HSPs, heat shock proteins; INT, introgression; KO, knockout; OE, overexpression; QTL, quantitative trait locus; ROS, reactive oxygen species; SKO, single knockout mutants; TF, transcription factor.

### 5.4 Maintaining protein stability under heat stress

Elevated temperatures disrupt protein homeostasis in plant cells by interfering with processes such as synthesis, folding, quality control, and intracellular transport (Peer et al., 2020). As a result, improperly folded or misfolded proteins accumulate, which can be detrimental to cellular function. When these defective proteins accumulate in the endoplasmic reticulum, the cell activates the unfolded protein response (UPR) (Peer et al., 2020). UPR serves as a protective mechanism, promoting the correct folding and processing of proteins to restore cellular stability. In plants, UPR is governed by two primary pathways. One involves inositol‐requiring enzyme 1 (IRE1), which facilitates the unconventional splicing of *bZIP60* mRNA, and the other is controlled by the TFs, *bZIP17* and *bZIP28* (S. Zhang et al., 2017). In *Arabidopsis*, heat stress activates all three pathways, with IRE1, bZIP60, and bZIP28 working together to maintain protein stability (Malini et al., 2020). Plants with mutations in these pathways, such as *bzip28 bzip60* double mutant or *bzip28* single mutant, exhibit increased sensitivity to heat, highlighting the essential role of the UPR in building heat tolerance (S. Zhang et al., 2017).

The UPR is a conserved cellular mechanism across plant species. For example, in maize (*Zea mays L*.), mutants lacking functional *bZIP60* are more sensitive to heat, underscoring the critical role of UPR in stress resilience (Z. Li et al., 2020). In rice, exposure to high temperatures triggers alternative splicing of *OsbZIP74* and *OsbZIP50* homologs of *bZIP60*‐producing nuclear‐targeted protein variants that activate key UPR genes such as *BiPs*, *PDIL1‐1*, *Calnexin*, and *SAR1B‐like* (Cheng et al., 2015). Heat stress also induces the nuclear translocation of *OsNTL3*, a transcriptional regulator upstream of *OsbZIP74* (X. H. Liu, Lyu, et al., 2020). Notably, overexpression of *OsNTL3* enhances heat tolerance in rice seedlings, emphasizing its regulatory importance. Furthermore, core UPR components such as *IRE1*, *bZIP28*, *bZIP17*, and the molecular chaperone *BiP1* are significantly upregulated during thermal stress, reaffirming their role of UPR signaling in restoring protein homeostasis under adverse conditions (Cheng et al., 2015).

### 5.5 HSPs and protein homeostasis

HSPs function as molecular chaperones, stabilizing unfolded proteins, preventing aggregation, and refolding polypeptides damaged by heat stress. By maintaining protein structure and turnover at elevated temperatures, HSPs are essential for preserving cellular protein homeostasis during the period of heat stress. HSPs are classified into six major groups based on their molecular size: small HSPs (sHSPs), HSP40, HSP60, HSP70, HSP90, and HSP100 (Jee, 2016). These protein families are strongly induced under heat stress, reflecting their critical role in enhancing cellular protection and stability. Notably, sHSPs are expressed at higher levels in heat‐resistant rice cultivars compared to heat‐susceptible ones, suggesting their potential as molecular markers for screening thermotolerant genotypes (Lin et al., 2014). Purified small HSPs exhibit robust chaperone activity, effectively inhibiting protein aggregation under stress conditions. The expression of HSP101, HSP90, and HSP70 increases significantly in rice following heat stress exposure, further supporting their crucial role in promoting thermotolerance (Lin et al., 2014). When rice plants are exposed to extreme heat stress, misfolded and aggregated proteins can accumulate to levels that exceed the cell's capacity for repair, necessitating their degradation rather than refolding. The ubiquitin/26S proteasome pathway is a vital proteolytic system that targets and degrades proteins marked with ubiquitin. In rice, multiple ubiquitin E3 ligases containing RING finger domains, including *OsHIRP1*, heat tolerance associated small protein (*OsHTAS*), and *OsHCI1*, play key roles in the selective removal of misfolded proteins, contributing significantly to heat stress tolerance (Lim et al., 2013). Additionally, *OgTT1*, a 26S proteasome α2 subunit cloned from *Oryza glaberrima* (African rice), exhibits superior efficiency in removing cytotoxic, denatured proteins compared to its *O. sativa* (Asian rice) homolog, *OsTT1* (X.‐M. Li, Chao, et al., 2015). This suggests that the proteasomal machinery in *O. glaberrima* has evolved to provide greater resilience against heat stress‐induced protein damage. *OsHIRP1*, *OsHTAS*, *OsHCI1*, and *OsTT1* are all heat‐inducible, and their overexpression substantially improves thermotolerance in rice (Kim et al., 2019). This highlights the crucial role that protein degradation plays in the plant's adaptation to heat stress (Lim et al., 2013).

### 5.6 Translational regulation and protein homeostasis during heat stress

Maintaining protein stability under heat stress relies heavily on precise regulation of the translation process, which requires the coordinated processing of mRNA, tRNA, and rRNA. *AET1*, a tRNA His guanylyl transferase, is essential for maintaining global tRNA balance and ensuring efficient translation under high‐temperature conditions. Mutations in *AET1* result in a pronounced thermosensitive phenotype, likely due to impaired translational capacity and reduced synthesis of essential growth‐related proteins (Chen et al., 2019). Similarly, tRNA thiolation is vital for maintaining proteostasis during heat stress. Mutations in the tRNA thiolation protein gene *SLG1* lead to chronic proteotoxic stress, increasing heat sensitivity and reducing plant survival (Y. Xu, Zhang, et al., 2020). The RNA 5‐methylcytosine (m5C) methyltransferase *OsNSUN2* is crucial for the proper accumulation of proteins related to photosynthesis and detoxification during heat stress. Knockout mutants of *OsNSUN2* display severe heat sensitivity, highlighting the importance of RNA modifications in translational regulation (Y. Tang et al., 2020). Additionally, rRNA homeostasis is fundamental for maintaining translation efficiency during heat stress. The RNA helicase *TOGR1* (thermo‐sensitive Obg‐like GTPase 1) plays a key role in stabilizing rRNA molecules at high temperatures. Overexpression of *TOGR1* enhances heat tolerance, supporting sustained growth and development in rice under heat stress conditions (D. Wang et al., 2016).

## 6 EPIGENETIC REGULATION OF HEAT STRESS ADAPTATION

Plants, like sessile organisms, frequently encounter recurring episodes of environmental stress throughout their life cycle. To overcome these challenges, they have evolved memory systems that enable more effective response to repeated stimuli. Recent research has identified changes in chromatin structure, adjustments in nucleosome arrangement, and changes in DNA methylation as fundamental components of these adaptive mechanisms. Epigenetic alterations play a significant role in stress memory, allowing plants to mount faster and stronger defense responses when reexposed to similar stress conditions (Oberkofler et al., 2021). Additionally, these modifications can be inherited by the offspring of stressed plants, a phenomenon known as transgenerational stress memory. This inheritance contributes to genetic diversity and provides an evolutionary advantage by facilitating adaptation to heat stress at the population level (Stief et al., 2014).

### 6.1 Chromatin dynamics and epigenetic regulation during heat stress memory

Plants encounter a wide array of both biotic and abiotic challenges in their natural habitat. Plants have evolved stress memory mechanisms that allow them to respond more effectively to repeated stress events. Stress memory involves a sequence of events: an initial environmental signal is detected, stored at the molecular level, and later retrieved when the same stress is encountered again. Chemical modifications to histone proteins such as methylation, acetylation, phosphorylation, and ubiquitination play a crucial role in reshaping chromatin architecture and regulating gene expression. Notably, trimethylation at lysine 4 of histone H3 is widely associated with active transcription in both plants and animals (Lämke et al., 2016). Sustained H3K4 methylation at heat‐inducible genes like *HSP18.2*, *HSP21.9*, and *HSP22.0* serves as an epigenetic marker of heat stress memory in *Arabidopsis* (Lämke et al., 2016) These modifications enhance the reactivation of heat shock genes upon repeated heat stress exposure, thereby improving plant resilience. HSFA2 is a key TF involved in recruiting H3K4me2/me3 marks to memory‐associated loci, ensuring prolonged gene expression and similar epigenetic roles are suggested in rice, although direct functional evidence remains limited (Brzezinka et al., 2016). Interestingly, in mammals, HSFA1 plays a similar role in modulating chromatin structure under stress, suggesting that TF‐mediated epigenetic regulation of heat stress responses is a conserved mechanism across eukaryotes (Ohama et al., 2017). In addition to H3K4 methylation, nucleosome repositioning is essential for maintaining heat stress memory. In *Arabidopsis*, the anti‐silencing function 1 (ASF1) protein homologs, AtASF1A and AtASF1B, contribute to the activation of HSPs and HSFs and while comparable chromatin remodeling mechanisms are proposed in rice, their functional characterization is still emerging. (Weng et al., 2014). Recruitment of ASF1A/B to chromatin correlates with a decrease in nucleosome density at gene promoters, improving accessibility for the transcriptional machinery and enhancing gene expression. ASF1A and ASF1B also promote histone H3 acetylation at lysine 56 (H3K56ac), which boosts the expression of key heat‐responsive genes like *HSFA2* and *HSFA3* during thermal stress (L. Wang et al., 2014). Further evidence for the role of nucleosome remodeling in heat stress memory comes from the *Arabidopsis* gene *Forgetter1* (*FGT1*). This gene interacts with chromatin‐remodeling proteins such as BRM, CHR11, and CHR17 components of the SWI (SWItch)/SNF (Sucrose Non‐Fermenting) and ISWI (Imitation SWItch) complexes and facilitates nucleosome displacement at the transcription start sites of genes involved in heat stress memory, enabling their rapid reactivation upon subsequent heat stress exposure and although homologs exist in other species, their functional roles in rice heat stress memory remain to be fully elucidated (Song et al., 2021). Interestingly, homologs of *FGT1* have been identified in metazoans, suggesting a broader functional role beyond heat stress memory. However, the precise connection between *FGT1* and H3K4 hypermethylation, as well as its involvement in other stress memory pathways, remains an area of active research (Brzezinka et al., 2016).

Much of the current understanding of small RNA‐mediated heat stress responses and memory is derived from *A. thaliana*, and although similar pathways are reported in rice, functional validation remains limited for several components.

### 6.2 Small and long noncoding RNA‐mediated control of plant heat stress responses and memory

Small RNAs, particularly microRNAs, are key regulators of TFs involved in heat stress responses and have recently been recognized as vital components of heat stress memory (Farooq et al., 2025). The *miR156* pathway, which includes associated proteins such as ARGONAUTE1, DICER‐LIKE1, and SUO, is implicated in the maintenance of heat stress memory in *Arabidopsis* (Stief et al., 2014). Heat stress induces a significant upregulation of *miR156*, which extends heat stress memory by suppressing *squamosa‐promoter binding protein‐like* (*SPL*) genes. In addition to its developmental role, miR156 is thought to bridge growth and stress response processes through the *miR156‐SPL* signaling module (Stief et al., 2014). Another key microRNA, *miR398*, is crucial for managing ROS levels during heat stress. Research shows that *miR398* downregulates genes encoding ROS‐detoxifying enzymes, such as Cu/Zn superoxide dismutases (*CSD1* and *CSD2*) and the copper chaperone CCS, thereby influencing oxidative stress responses (Guan et al., 2013). Heat stress strongly upregulates *miR398*, leading to the suppression of *CSD1* and *CSD2*, which causes ROS levels to rise. This ROS accumulation activates *HSFA1*, creating a positive feedback loop that intensifies the heat stress response and enhances plant thermotolerance (Guan et al., 2013). Beyond microRNAs, trans‐acting small interfering RNAs also participate in plant responses to heat stress. In *Arabidopsis*, the heat‐induced *trans‐acting SiRNA Precursor 1* (*TAS1*) is a key player in thermotolerance, functioning within the HSFA1a signaling pathway. *TAS1* regulates the heat response by targeting *HTT1* and *HTT2* genes, whose encoded proteins interact with HSPs such as Hsp70‐14 and Hsp40, as well as the TF NF‐YC2, thereby promoting heat stress tolerance (S. Li et al., 2014). These interactions result in complexes that bolster the plant's thermotolerance. Overexpression of *HTT1* and *HTT2* increases heat tolerance, establishing these genes as downstream effectors of *HSFA1a* (Guan et al., 2013). The role of tocopherols (Vitamin E) is highlighted for modulating miRNA biogenesis. Tocopherols contribute to the accumulation of 3′‐phosphoadenosine 5′‐phosphate, an inhibitor of exoribonuclease (XRN)‐mediated primary miRNA degradation. This stabilization of miRNAs under heat stress enhances heat stress tolerance, indicating a chloroplast‐to‐nucleus retrograde signaling mechanism that integrates chloroplast function with miRNA‐mediated heat stress adaptation (X. Fang et al., 2019). Additionally, alternative splicing has emerged as a key factor in heat stress memory, allowing plants to preserve molecular information from past heat stress events. By generating specific transcript isoforms, alternative splicing enables plants to mount a more rapid and efficient response to subsequent heat stress episodes. This mechanism provides a molecular basis for transcriptional memory, ensuring that plants are better prepared for future stress conditions (Sanyal et al., 2018).

Genome‐wide transcriptome analysis in rice has identified several heat‐responsive long noncoding RNAs (HRLs) that participate in thermal adaptation. Key HRLs such as TCONS_00018499, TCONS_00018800, TCONS_00018917, and TCONS_00018921 are located within major heat‐tolerance quantitative trait loci (QTLs) and show strong differential expression under high‐temperature conditions (Z. Zhang, Zhong, et al., 2022). Additional HRLs, including TCONS_00018455, TCONS_00077715, TCONS_00043075, and TCONS_00092993, function through cis‐regulation or by forming ceRNA modules with heat‐responsive miRNAs to modulate downstream stress genes (Z. Zhang, Zhong, et al., 2022). qRT‐PCR validation further confirmed the heat‐induced expression of TCONS_00070624, TCONS_00088405, and TCONS_00100154, reinforcing their roles in the rice heat stress regulatory network. Collectively, these HRLs form a multilayered regulatory system linking lncRNA expression, miRNA activity, and heat‐responsive gene regulation in rice (Z. Zhang, Zhong, et al., 2022).

Several studies in rice provide increasing evidence for the relevance of these regulatory mechanisms under heat stress. Recent advances have begun to reveal gene‐level evidence of epigenetic memory associated with heat stress, particularly in crop species such as rice. Although much of the mechanistic understanding derives from *Arabidopsis* (Brzezinka et al., 2016; Lämke et al., 2016), similar principles have also been demonstrated in cereals through DNA methylation‐mediated transcriptional regulation. For example, heat stress during grain filling in rice induces stable changes in promoter methylation of key starch metabolism genes, including *OsAGPS2b*, *OsGBSSI*, and *OsSuSy2*, resulting in enhanced expression in subsequent generations (Table 3).

**TABLE 3:** Key genes involved in epigenetic memory under abiotic stress, highlighting chromatin modifications, RNA‐mediated regulation, and DNA methylation‐based transgenerational memory mechanisms in model plants and rice.

Gene	Species	Epigenetic mechanism	Role in stress memory	Stress specificity
*HSFA2*	*Arabidopsis thaliana*	H3K4me2/3 histone methylation	Maintains transcriptional memory of heat‐responsive genes	Heat
*HSP18.2*/*HSP21*	*Arabidopsis thaliana*	H3K4me3 enrichment	Sustained activation upon repeated heat exposure	Heat
*FGT1* (*Forgetter1*)	*Arabidopsis thaliana*	Chromatin remodeling (SWI/SNF complex)	Enables rapid reactivation of memory‐associated genes	Heat
*ASF1A*/*ASF1B*	*Arabidopsis thaliana*	Histone chaperone (H3K56 acetylation)	Promotes nucleosome eviction and transcriptional memory	Heat
*miR156*	*Arabidopsis thaliana*	miRNA‐mediated regulation	Extends stress memory via repression of SPL transcription factors	Heat/drought
*miR398*	*Arabidopsis thaliana*	miRNA‐mediated ROS regulation	Modulates ROS signaling and reinforces heat response memory	Heat/oxidative stress
*TAS1–HTT1/HTT2 module*	*Arabidopsis thaliana*	ta‐siRNA pathway	Enhances thermotolerance via posttranscriptional regulation	Heat (also general stress)
*ONSEN*	*Arabidopsis thaliana*	Transposon activation	Generates heritable stress‐responsive epigenetic variation	Heat
*DDM1/MOM1*	*Arabidopsis thaliana*	DNA methylation maintenance	Restores epigenetic state after stress and regulates memory stability	General abiotic stress
*OsAGPS2b*	*Oryza sativa*	Promoter hypomethylation	Enhances starch biosynthesis gene expression in progeny	Heat
*OsGBSSI*	*Oryza sativa*	Promoter hypomethylation	Improves grain filling and reduces chalkiness under repeated heat stress	Heat
*OsSuSy2*	*Oryza sativa*	Promoter hypomethylation	Maintains carbohydrate metabolism during heat stress memory	Heat
*OsAmy1A/OsAmy3D*	*Oryza sativa*	Promoter hypermethylation	Suppresses starch degradation, improving grain quality in progeny	Heat

In contrast, the promoters of starch‐degrading genes such as *OsAmy1* and *OsAmy3D* undergo hypermethylation, leading to transcriptional repression and improved grain quality under recurrent heat stress. These findings highlight a direct link between epigenetic reprogramming and adaptive phenotypic responses, demonstrating that DNA methylation plays a central role in transgenerational heat stress memory in rice (Suriyasak et al., 2025). Together with well‐characterized regulators identified in model systems, these rice‐specific genes provide important insights into how epigenetic mechanisms may be leveraged to improve thermotolerance in crop plants.

### 6.3 Inheritance of heat stress memory across plant generations

Although most heat stress memory mechanisms in plants are temporary and revert to baseline physiological states a few days after stress subsides, some plants exhibit transgenerational heat stress memory. This phenomenon allows certain stress‐induced molecular changes to persist across generations, providing a potential evolutionary advantage in fluctuating environmental conditions. Transposable elements (TEs) are widely distributed throughout eukaryotic genomes. Because of their potential mutagenic effects, plants have evolved sophisticated transcriptional and posttranscriptional regulatory mechanisms to tightly control TE activity. Under stress conditions, plants may temporarily relax transposon silencing, allowing for transient TE activation. However, in most cases, the epigenetic landscape returns to its prestress state once the stress is alleviated (Lang‐Mladek et al., 2010). A key regulator of chromatin architecture in response to heat stress is *Heat‐Intolerant 4* (*HIT4*), which facilitates the decondensation of heterochromatin during heat stress, leading to the temporary activation of certain repetitive DNA sequences (Perrella et al., 2022). This chromatin relaxation enhances basal thermotolerance, enabling plants to adapt to extreme temperatures. Once the stress subsides, chromatin is restored to its prestress configuration, preserving genomic stability while ensuring adaptability under future stress (L. C. Wang et al., 2013). The chromatin remodelers *Decrease in DNA Methylation 1* (*DDM1*) and *Morpheus Molecule 1 (MOM1)*, play crucial roles in reestablishing epigenetic silencing after heat stress (Torres & Sanchez, 2024). Studies in *Arabidopsis* have shown that the *Copia‐type retrotransposon ONSEN* becomes transcriptionally active under heat stress (Cavrak et al., 2014). This activation results in the production of extrachromosomal DNA copies in mutants lacking proper small RNA biogenesis. In normal plants, *ONSEN* transcripts and extrachromosomal DNA are no longer detectable 20–30 days after heat stress. However, the progeny of heat‐stressed mutants displays a higher occurrence of new *ONSEN* insertions, indicating likely *ONSEN* transposition during flower development, prior to gametogenesis. Mutant plants that lack efficient siRNA‐mediated silencing mechanisms retain stress memory throughout their development, allowing *ONSEN* to remain active and transpose during reproductive organ formation. In contrast, this phenomenon is absent in wild‐type plants, highlighting the crucial role of the siRNA pathway in suppressing transposon reactivation triggered by environmental stress. Natural or experimentally induced variation in *ONSEN* insertions can impart heat responsiveness to adjacent genes, establishing new heat stress‐responsive regulatory networks (Ito et al., 2011). Further studies have revealed that *ONSEN* activation under heat stress depends on HSFs, as its long terminal repeat contains HSEs (Cavrak et al., 2014). Both HSFA2 and HSFA1s are essential for the heat‐induced activation of *ONSEN*, demonstrating that transposons can exploit the plant's heat stress response for their own activation. Conversely, plants may use this stress‐induced transposition to establish new regulatory networks that enhance adaptation to heat stress, illustrating a dynamic interplay between genome evolution and environmental stress adaptation (Ito et al., 2011).

Heat stress has been found to induce a regulatory feedback loop involving *HSFA2*, which promotes early flowering and modifies immune responses in the progeny of heat‐exposed *Arabidopsis* plants (J. Liu et al., 2019). Under heat stress, *HSFA2* is activated and directly upregulates *REF6*, an H3K27me3 demethylase associated with flowering time regulation. *REF6*, in turn, enhances HSFA2 expression, creating a self‐sustaining regulatory loop that can be inherited across generations. This feedback loop involving *HSFA2* and *REF6* is crucial for preserving the long‐term epigenetic memory of heat stress, allowing plants to retain and utilize past stress information. Plants exposed to high temperatures for one generation exhibit increased thermotolerance and altered developmental patterns in the next generation (J. Liu et al., 2019).

### 6.4 Transcriptional reprogramming following heat stress

Although considerable progress has been made in understanding heat stress memory, the mechanisms underlying its dissipation remain less defined. In *Arabidopsis*, autophagy plays a central role in resetting cellular homeostasis following heat stress (Sedaghatmehr et al., 2019). Thermopriming induces sustained autophagy during recovery, promoting the degradation of HSPs and other stress‐associated proteins. The ubiquitin‐like protein ATG8 mediates selective targeting of HSPs, including HSP17.6, HSP101, and HSP90, for autophagic degradation, facilitating thermorecovery within several days post‐stress. Inhibition or loss of autophagy delays HSP turnover, leads to protein aggregation and increased ubiquitination, and prolongs stress‐responsive gene expression. These findings indicate that autophagy acts as a negative regulator of heat stress memory, restoring proteome balance and redirecting resources toward normal growth. Together, autophagy integrates with epigenetic and transcriptional regulation to balance stress adaptation and developmental progression.

Importantly, epigenetic mechanisms, including DNA methylation, histone modifications, and small RNA‐mediated regulation, contribute to phenotypic plasticity and stress adaptation in rice. These regulatory layers modulate the expression of genes associated with heat tolerance and contribute to phenotypic variation captured in GWAS and GS models. Accordingly, integrating epigenetic information with genomic data offers valuable opportunities to improve trait‐mapping resolution and predictive accuracy in breeding programs (Crossa et al., 2017; Springer & Schmitz, 2017).

## 7 STRATEGIES FOR ENHANCING HEAT TOLERANCE

### 7.1 Detection of heat‐tolerant QTLs and marker‐assisted breeding

Advances in molecular and physiological studies (Sections 3, 4, 5, 6) have identified key genes, regulatory pathways, and epigenetic mechanisms underlying heat stress responses in rice. These findings provide a strong foundation for translating biological insights into genomic and breeding strategies. For example, genes associated with reproductive thermotolerance, stress signaling, and grain quality regulation constitute important targets for genomic analysis. Such candidate genes can be integrated into QTL mapping and GWAS to identify trait‐associated loci, which can subsequently be incorporated into GS models for predicting breeding values. Moreover, the integration of gene‐level information with genomic prediction and multi‐environment data can enhance the efficiency of breeding programs aimed at improving heat resilience (Varshney et al., 2021).

**FIGURE 6:**
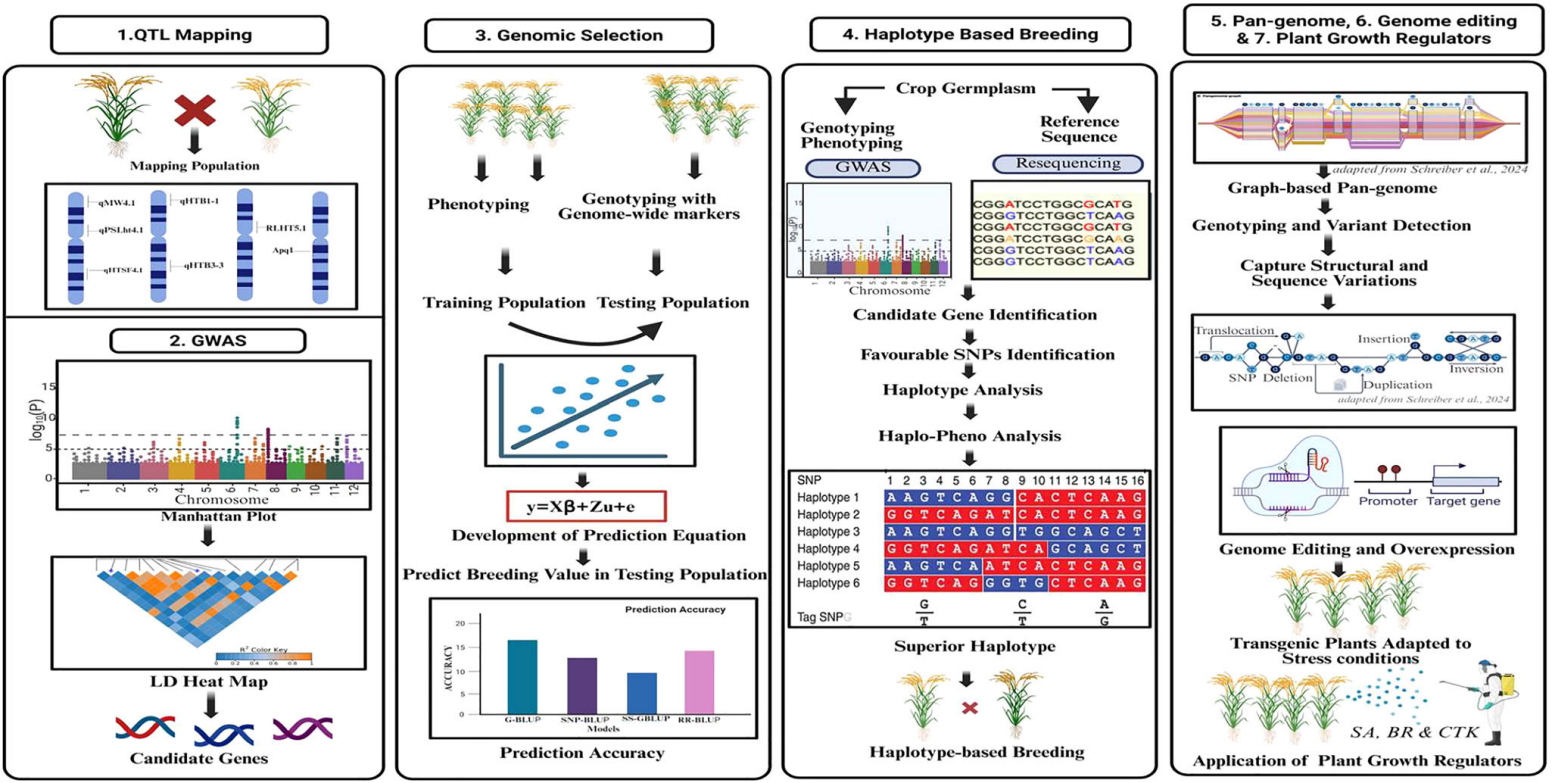
Stepwise framework integrating genomic and breeding strategies for developing heat‐resilient rice cultivars. The figure illustrates a sequential pipeline from genetic discovery to advanced breeding and management interventions for improving heat tolerance in rice. (1) *Quantitative trait loci* (*QTL*) *mapping*: Identification of genomic regions associated with heat tolerance using biparental mapping populations and linkage analysis. This approach detects major‐effect loci controlling traits such as spikelet fertility, grain yield, and stress responsiveness. (2) *Genome‐wide association studies* (*GWAS*): High‐resolution identification of marker–trait associations using diverse germplasm panels and genome‐wide single nucleotide polymorphism (SNP) data. GWAS enables fine mapping of loci associated with complex traits, as visualized through Manhattan plots and linkage disequilibrium (LD) patterns, leading to candidate gene identification. (3) *Genomic selection* (*GS*): Implementation of genome‐wide prediction models, including genomic best linear unbiased prediction (GBLUP), ridge regression BLUP (RR‐BLUP), ssGBLUP, and SNP‐BLUP, to estimate breeding values by capturing the cumulative effects of small‐effect alleles. Training and testing populations are used to develop prediction equations, enabling accelerated selection for heat tolerance traits. (4) *Haplotype‐based breeding*: Integration of genotyping and phenotyping data to identify favorable haplotypes and superior allele combinations. This approach improves the precision of selection and facilitates the introgression of thermotolerance traits into elite cultivars through haplotype‐informed breeding strategies. (5) *Pangenome and graph‐based genomics*: Use of graph‐based pan‐genome frameworks to capture structural and sequence variation, including insertions, deletions, duplications, and inversions, which are often missed by single‐reference genome approaches. This enhances the identification of novel alleles associated with stress adaptation. (6) *Genome editing and transgenic approaches*: Application of targeted genome editing technologies (e.g., CRISPR [Clustered Regularly Interspaced Short Palindromic Repeats]/Cas) and gene overexpression strategies to precisely modify candidate genes associated with heat tolerance, enabling functional validation and trait improvement. (7) *Application of plant growth regulators*: Use of growth regulators such as salicylic acid (SA), brassinosteroids (BR), and cytokinins (CTK) to modulate physiological responses and enhance plant resilience under heat stress conditions in field environments.

Given the limitations of conventional breeding and agronomic strategies, identification and deployment of QTLs associated with heat tolerance have become central to rice improvement programs. Thermotolerance in rice is a complex, polygenic trait influenced by multiple loci with stage‐specific and environment‐dependent effects. Numerous QTLs governing heat response have been mapped across developmental stages, particularly during the reproductive phase, when elevated temperatures severely compromise spikelet fertility, grain filling, and ultimately yield stability. Collectively, the major heat‐responsive QTLs identified across developmental stages are summarized in Table 4, and their integration into modern breeding and genomic strategies is illustrated in Figure 6.

**TABLE 4:** Major quantitative trait loci (QTLs) associated with heat tolerance in rice.

QTL/gene	Chr	Growth stage	Associated trait	Functional relevance	Reference
*RLHT5.1*	5	Seedling	Root length under heat	Regulates root elongation under high temperature	(M. Li, Li, et al., 2018)
*qHTB1‐1*	1	Booting	Spikelet fertility	Stable across generations; applied in marker‐assisted selection	(M. Li, Li, et al., 2018)
*qHTB3‐3*	3	Booting	Reproductive tolerance	Located near thermotolerance loci; enhances fertility stability	(M. Li, Li, et al., 2018)
*qHTSF1.1*	1	Reproductive	Spikelet fertility	Major‐effect locus for fertility under heat stress	(Ye et al., 2015)
*qHTSF4.1*	4	Reproductive	Spikelet fertility	Fine‐mapped; overlaps thermotolerance‐associated regions	(Ye et al., 2015)
*qPSLht4.1*	4	Anthesis	Flowering‐stage tolerance	Validated under multiple heat regimes	(Xiao et al., 2011)
*qHTH5*	5	Heading/flowering	Reproductive tolerance	Fine‐mapped to 304.2 kb; wild‐derived allele	(C. Hu et al., 2022)
*qHTH10*	10	Heading	Heat tolerance	Novel wild‐derived thermotolerance locus	(C. Hu et al., 2022)
*qSTIPSS9.1*	9	Reproductive	Spikelet sterility	Strong‐effect allele (<400 kb interval)	(Shanmugavadivel et al., 2017)
*qSTIY5.1*/*qSSIY5.2*	5	Reproductive	Sterility/yield stability	Strong‐effect fertility QTL	(Shanmugavadivel et al., 2017)
*qEMF3*	3	Flowering	Early‐morning flowering	Heat avoidance via anthesis time shift	(Hirabayashi et al., 2015)
*Apq1* (*OsSus3 allele*)	7	Grain filling	Grain appearance	Links sucrose metabolism to thermotolerance	(Takehara et al., 2018)
*qPGC5*	5	Grain filling	Chalkiness	Regulates heat‐induced grain chalkiness	(Yang et al., 2022)
*qPGC6*	6	Grain filling	Chalkiness	Stabilizes grain appearance under heat	(Yang et al., 2022)
*qHAC4*	4	Grain filling	Amylose content	Modulates Wx gene splicing efficiency	(H. Zhang et al., 2014)
*qHAC8a*	8	Grain filling	Amylose content	Stabilizes starch biosynthesis under heat	(H. Zhang et al., 2014)
*qHAC8b*	8	Grain filling	Amylose content	Enhances starch pathway resilience	(H. Zhang et al., 2014)
*qHAC10*	10	Grain filling	Amylose content	Improves grain quality stability	(H. Zhang et al., 2014)
*qWB6*	6	Grain quality	Grain appearance	Improves grain quality stability under heat stress	(Xiao et al., 2011)
*qWB8*	8	Grain quality	Grain appearance	Enhance grain quality under high temperature	(Xiao et al., 2011)
*qWB9*	9	Grain quality	Grain quality	Confers improved grain resilience under heat	(Xiao et al., 2011)
*qMW4.1*	4	Grain quality	Grain stability	Validated across environments; improves quality under stress	(Cao et al., 2020)

Heat tolerance QTLs have been identified across multiple developmental stages. At the seedling stage, *RLHT5.1* on chromosome 5 regulates root elongation under high‐temperature stress, contributing to improved early vigor and enhanced establishment under thermal stress conditions (M. Li, Li, et al., 2018). Several reproductive‐stage QTLs associated with spikelet fertility and seed‐setting under high temperature have been consistently identified across diverse mapping populations. For instance, *qHTB1‐1* and *qHTB3‐3* (booting stage) exhibited stable phenotypic effects across genetic backgrounds, suggesting moderate‐to‐major genetic contributions to reproductive thermotolerance (M. Li, Li, et al., 2018). Likewise, *qHTSF1.1* and *qHTSF4.1*, mapped using IR64/N22 populations, explained substantial variation in spikelet fertility under heat stress and represent foundational loci for reproductive‐stage resilience (Ye et al., 2015). Furthermore, *qPSLht4.1*, mapped to chromosome 4, confers tolerance specifically during anthesis and has been validated under multiple heat regimes, highlighting its importance in minimizing flowering‐stage sterility (Xiao et al., 2011). Importantly, the detection of these QTLs across independent studies indicates partial conservation of thermotolerance mechanisms within elite germplasm pools.

Wild rice germplasm has further expanded the thermotolerance QTL landscape. Loci such as *qHTH5* and *qHTH10*, identified from wild rice‐derived populations, provide novel allelic diversity absent in cultivated backgrounds (C. Hu et al., 2022). The fine‐mapping of *qHTH5* to a 304.2 kb interval significantly enhances its breeding utility and facilitates candidate gene discovery. Similarly, spikelet sterility QTLs including *qSTIPSS9.1* and *qSTIY5.1/qSSIY5.2*, localized within genomic regions smaller than 400 kb, suggest the presence of relatively strong‐effect alleles that may underlying critical reproductive stability mechanisms under high‐temperature conditions (Shanmugavadivel et al., 2017). These loci are particularly valuable because reproductive‐stage heat sensitivity remains the primary constraint on rice productivity in warming climates.

Beyond reproductive stability, grain quality maintenance under heat stress has emerged as a parallel breeding priority. Substitution mapping approaches identified *qPGC5* and *qPGC6*, which regulate heat‐induced chalkiness, a major determinant of milling recovery and consumer acceptance (Yang et al., 2022). Additional grain‐appearance QTLs, including *qWB6*, *qWB8*, and *qWB9*, have also been reported to enhance grain quality stability under heat stress by improving grain transparency and structural integrity during high‐temperature (Xiao et al., 2011). Similarly, *qMW4.1* contributes to improved grain weight and quality stability under elevated temperatures and has demonstrated relatively consistent performance across multiple environments, indicating its potential utility in marker‐assisted breeding programs (Cao et al., 2020). In parallel, amylose‐content‐associated loci *qHAC4*, *qHAC8a*, *qHAC8b*, and *qHAC10* were shown to mitigate heat‐induced deterioration of grain quality through modulation of *Wx* gene splicing efficiency, thereby stabilizing starch biosynthesis pathways under elevated temperature (H. Zhang et al., 2014). These findings highlight that thermotolerance breeding must integrate both yield stability and grain quality preservation, as the latter significantly influences economic returns.

Advancing flowering to the early morning, when temperatures are lower, is an effective strategy to reduce heat induced spikelet sterility in rice (Sheehy et al., 2005). This distinct heat avoidance mechanism is illustrated by *qEMF3*, introgressed from a wild species *Oryza officinalis*, which advances flower opening time to early morning hours, thereby enabling plants to escape peak daytime heat stress (Hirabayashi et al., 2015). However, under extreme temperatures, early flowering genotypes may still suffer substantial fertility loss, indicating that phenological escape alone is insufficient for full thermotolerance (Ishimaru et al., 2010). The incorporation of thermotolerant loci that shift peak anthesis timing can further mitigate heat exposure, though their stability and agronomic performance in tropical environments remain uncertain (Hirabayashi et al., 2015).

Despite extensive QTL discovery, only a subset of loci has been cloned and functionally characterized. Among these, *TT1* (*Thermo Tolerance 1*) enhances seedling thermotolerance by maintaining chloroplast integrity and proteostasis (X.‐M. Li, Chao, et al., 2015). *TT2*, encoding a heterotrimeric G‐protein γ‐subunit, modulates Ca^2^^+^ signaling and wax metabolism, thereby conferring tolerance at both vegetative and reproductive stages (Kan et al., 2023). *SLG1* contributes to thermotolerance through cytoskeletal regulation, affecting cellular organization under heat stress (Kobayashi et al., 2013). Similarly, *OsHTAS*, cloned from chromosome 9, improves seedling survival under extreme heat by enhancing antioxidant capacity and ROS homeostasis (Kilasi et al., 2018). At the grain‐filling stage, *Apq1*, corresponding to a thermo‐responsive allele of *OsSus3*, links sucrose metabolism with improved grain appearance and quality under high temperature (Takehara et al., 2018). These cloned loci provide mechanistic validation of QTL effects and enable precise marker‐assisted or gene‐based introgression. Collectively, the identified QTLs reveal that rice thermotolerance involves multiple biological pathways, including reproductive organ development, carbohydrate metabolism, starch biosynthesis, ROS detoxification, Ca^2^^+^‐mediated signaling, phenological adjustment, and proteostasis maintenance. However, the majority of mapped QTLs remain uncharacterized at the gene level, and their stability across diverse agroecological environments is not fully established. Genotype × environment interactions often reduce QTL consistency, limiting their direct transferability. Therefore, future breeding strategies should integrate high‐resolution fine mapping, allele mining in wild and landrace germplasm, multi‐environment validation, and pyramiding of nonallelic QTLs with complementary physiological functions. Combining reproductive‐stage fertility loci with grain‐quality‐stabilizing and heat‐avoidance QTLs will be essential to achieve durable thermotolerance under progressive climate warming (Cao et al., 2020).

Effective translation of thermotolerance into breeding outcomes requires the definition of key target traits and physiological thresholds to guide selection and genomic prediction. Reproductive‐stage spikelet fertility under high temperature remains the most critical trait, particularly under exposure to temperatures ≥35°C during anthesis, because it directly determines yield stability (Jagadish et al., 2007; Prasad et al., 2006). Grain quality traits, including chalkiness and amylose content under elevated temperature, should also be prioritized because they influence market value and processing quality (Folsom et al., 2014; Wada et al., 2015). In addition, physiological traits such as canopy temperature regulation, membrane stability, and maintenance of photosynthetic efficiency under heat stress provide valuable secondary selection criteria (Bita & Gerats, 2013; Sailaja et al., 2015). From a breeding perspective, these traits should be assessed under clearly defined heat regimes that distinguish between acute stress during flowering and prolonged stress during grain filling. Incorporating such trait‐specific thresholds into GS models, haplotype‐based breeding, and QTL pyramiding strategies will improve predictive accuracy and facilitate the development of rice cultivars with stable performance across diverse heat‐stress environments (Cao et al., 2020; Ye et al., 2015).

### 7.2 Genome‐wide association studies

GWAS are powerful genomic tools used to identify statistical associations between genetic variants typically single nucleotide polymorphisms (SNPs) and phenotypic traits across large and genetically diverse populations (Uffelmann et al., 2021). Unlike traditional biparental QTL mapping, which relies on limited recombination within a single cross, GWAS leverages historical recombination events accumulated over many generations, enabling high‐resolution mapping of trait‐associated loci across the entire genome. This makes GWAS particularly valuable for dissecting complex quantitative traits such as heat tolerance, which are controlled by multiple small‐effect genes acting across various developmental stages.

In rice, GWAS has become indispensable for uncovering the natural allelic diversity underlying thermotolerance. By screening extensive germplasm panels exposed to high‐temperature stress during seedling, reproductive, and grain‐filling stages, GWAS has identified numerous reproducible loci associated with traits such as seedling survival, spikelet fertility, pollen viability, and grain quality parameters like chalkiness (Lafarge et al., 2017). Notable loci include *qHTT4.2* (Pan et al., 2023), as well as several grain‐appearance QTL clusters and candidate genes influencing heat‐induced grain chalkiness and pollen viability (P. Li et al., 2023; Ravikiran et al., 2022). Importantly, haplotype‐level analyses derived from GWAS enable breeders to identify superior allelic variants rather than relying solely on linked markers, thereby enhancing the precision of marker‐assisted selection (MAS). Because many GWAS‐detected loci demonstrate consistent effects across genetic backgrounds and environments (X. Huang et al., 2010), they serve as stable, field‐relevant thermotolerance loci that can be pyramided with known QTLs in elite germplasm. Collectively, GWAS bridges the gap between genetic discovery and applied breeding, providing a robust pipeline of validated markers, functional candidate genes, and targets for allele introgression or CRISPR (Clustered Regularly Interspaced Short Palindromic Repeats)‐based gene editing, expediting the development of heat‐resilient rice varieties suited for future climate challenges.

### 7.3 Genomic selection

GS has emerged as a powerful and predictive breeding tool for enhancing heat tolerance in rice. Unlike conventional MAS, which focuses on a few major loci, GS estimates genome‐wide marker effects to predict the breeding values of individuals, thereby capturing the polygenic architecture of thermotolerance (Spindel et al., 2015). This approach is particularly effective for complex traits like heat stress tolerance during flowering and grain‐filling stages, where collective actions of small‐effect alleles maintain yield stability. Recent studies in rice have demonstrated that incorporating high‐density SNP markers and diverse training populations significantly improves prediction accuracy and shortens selection cycles, enabling the early advancement of elite lines (Biswas et al., 2023; Spindel et al., 2015). For example, GS models trained on heat‐stressed phenotypes in populations derived from heat‐tolerant donors such as N22 and Giza178 achieved high predictive accuracy for spikelet fertility and grain yield under 38°C–40°C, allowing rapid identification of superior heat‐tolerant progenies (Spindel et al., 2015). Furthermore, GS models that incorporate physiological trait indices such as spikelet fertility, canopy temperature, and grain filling, along with multi‐environment data, improve prediction reliability across diverse climatic conditions (Spindel et al., 2015). However, the predictive accuracy of GS for heat tolerance remains constrained by the complex genetic architecture of thermotolerance‐related traits. Heat stress responses in rice are highly stage specific, with distinct genetic determinants underlying seedling vigor, reproductive‐stage fertility, and grain quality under elevated temperatures (Folsom et al., 2014; Jagadish et al., 2007). Reproductive‐stage traits, including pollen viability and spikelet fertility, are particularly sensitive to short‐duration heat events (≥35°C), yet they remain underrepresented in GS training datasets because of the difficulty of precise phenotyping under field conditions (Prasad et al., 2006). Consequently, many GS models rely on grain yield or correlated surrogate traits, which may not adequately capture the underlying biological responses to heat stress. In addition, thermotolerance exhibits strong genotype × environment (G × E) interactions, as variation in heat intensity, duration, and timing relative to developmental stage substantially influences trait expression and limits the transferability of prediction models across environments (Seneviratne et al., 2021). The genetic architecture of heat tolerance is also often nonlinear, particularly for threshold‐dependent traits such as spikelet sterility, in which small increases above critical temperatures can cause disproportionately large yield losses (C. Zhao et al., 2017). Conventional linear GS models, including genomic best linear unbiased prediction (GBLUP) and ridge regression BLUP (RR‐BLUP), may therefore have limited capacity to capture these complex relationships (Crossa et al., 2017). Overcoming these limitations will require the integration of stage‐specific phenotyping, high‐throughput phenomics, and environment‐aware GS models that explicitly account for G × E interactions, such as reaction‐norm models, together with nonlinear approaches including machine learning and deep learning frameworks capable of capturing complex trait architectures (Crossa et al., 2017; Montesinos‐López et al., 2018). Such advances will be essential for improving prediction accuracy and enabling the reliable deployment of thermotolerance traits in breeding programs. Overall, GS provides a scalable framework for pyramiding favorable alleles, minimizing linkage drag, and accelerating the development of climate‐resilient, heat‐tolerant rice cultivars.

A major and often underrecognized limitation in improving heat tolerance through genomic approaches is the lack of scalable, high‐resolution phenotyping. Accurate assessment of heat stress responses, particularly during reproductive stages such as anthesis, requires precise control of temperature, timing, and duration of stress exposure, which is difficult to achieve under field conditions (Araus & Cairns, 2014; Cobb et al., 2013). Traits such as pollen viability, spikelet fertility, and anther dehiscence are highly sensitive to short‐term heat events, yet remain challenging to quantify at scale, thereby limiting their inclusion in large breeding populations and GS models (Jagadish et al., 2007; Prasad et al., 2006). Consequently, most breeding programs rely on grain yield or surrogate traits, which may not fully reflect the underlying biological responses to heat stress. Addressing this bottleneck will require the integration of high‐throughput phenomics platforms, controlled‐environment screening systems, and remote‐sensing approaches capable of capturing dynamic stress responses across developmental stages (Araus & Cairns, 2014). Overcoming these phenotyping constraints is essential for improving genomic prediction accuracy and accelerating the development of heat‐resilient rice cultivars. In addition to its general framework, the effectiveness of GS for heat tolerance depends strongly on model choice, marker density, and training population design. Commonly used statistical models include GBLUP and RR‐BLUP, which assume uniform shrinkage of marker effects, as well as Bayesian approaches, such as BayesA, BayesB, and BayesCπ, that allow variable shrinkage and may better capture loci with moderate‐to‐large effects (Crossa et al., 2017; Meuwissen et al., 2001). Prediction accuracy is influenced by genome‐wide marker coverage and linkage disequilibrium with causal variants, although increasing marker density beyond an optimal threshold often yields diminishing returns (Spindel et al., 2015). Equally important is the composition of the training population, as genetic relatedness to the target population returns (Spindel et al., 2015). Equally important is the composition of the training population, as genetic relatedness to the target population and representation of relevant environmental conditions, particularly heat stress scenarios, determine model robustness. For complex traits such as thermotolerance, multi‐trait GS models that jointly analyze correlated traits, including pollen viability, spikelet fertility, grain yield, and canopy temperature, have demonstrated improved predictive performance by leveraging shared genetic architecture (Burgueño et al., 2012). Despite these advances, the practical deployment of GS for heat tolerance in rice remains constrained by the limited availability of large, well‐phenotyped populations under field‐relevant heat stress, challenges in capturing genotype × environment interactions, and the need for continuous model recalibration. Addressing these limitations will be essential for translating GS predictions into breeding gains under climate variability.

### 7.4 Haplotype‐based breeding

Traditional SNP‐based analyses fail to capture the combined effects of linked alleles, which are often essential for accurately predicting quantitative traits. This limitation highlights the need for haplotype‐based breeding approach, which is recognized as a transformative strategy for improving heat tolerance, as it enables the identification and selection of co‐inherited blocks of functional alleles that jointly regulate polygenic stress‐response pathways. This approach offers substantially greater predictive power than single‐SNP analyses (He et al., 2023). In rice, extensive resequencing efforts such as the 3K Rice Genomes Project, has enabled the identification of high‐resolution haplotypes associated with heat‐tolerance traits including spikelet fertility and panicle exertion (W. Wang, Mauleon, et al., 2018). Notably, haplotypes within the *qHTSF4.1* region have been linked to improved spikelet fertility under high day and night temperatures (Ye et al., 2015). Natural allelic variation in *HEADING DATE 1* (*Hd1*) and *DELAYED HEADING 1* (*DTH1*) has been associated with flowering‐time diversity and environmental adaptation (Hori et al., 2015; K. Zhao et al., 2011). The haplotype variation in *OsHSP101* and *OsHTAS* has been shown to contribute to tissue thermotolerance and ROS detoxification under acute heat episodes (Jagadish, Muthurajan, Oane, et al., 2010; J. Liu et al., 2016), highlighting the depth of functional diversity accessible within rice pangenomes.

Beyond rice, high‐resolution haplotype panels from major resequencing initiatives, such as the nested association mapping maize population, have identified haplotypes associated with pollen viability, canopy temperature regulation, and membrane stability under heat stress (Bukowski et al., 2017). In wheat (*Triticum aestivum L*.), haplotype dissection of the *TaHSP70* and *TaSST* regions has revealed allelic combinations linked to superior grain set and higher chlorophyll retention during temperature spikes (Sarwar et al., 2026). Similarly, in chickpea (*Cicer arietinum L*.), pangenome‐assisted haplotype analysis has detected SVs associated with oxidative stress mitigation and improved reproductive success under heat stress (Thudi et al., 2016). In sorghum (*Sorghum bicolor*), pangenome‐informed haplotypes identified key rearrangements influencing transpiration efficiency and high‐temperature tolerance (Morris et al., 2026). Collectively, these examples illustrate power of haplotype‐based approaches for deep understanding of genetic architecture underlying heat‐stress resilience across diverse crops.

Despite these advances, a major knowledge gap remains. Most identified haplotypes lack functional validation across diverse heat environments, and the mechanisms by which they influence reproductive resilience, pollen thermotolerance, or root water uptake under different heat intensities (Jagadish et al., 2021). This gap is particularly critical for rice, where flowering tissues are highly vulnerable to short‐duration heat spikes that significantly reduce yield potential (Jagadish et al., 2007;  ). Addressing these challenges will require integrated pipelines that combine pangenomic haplotype maps with multi‐omics profiling (transcriptomic, epigenomic, metabolomic), systems‐level genome‐to‐phenome modeling, and advanced heat phenotyping platforms. Such integration will be essential for identifying, validating, and deploying “causal haplotypes” in breeding pipelines, thereby accelerating the development of heat‐resilient cultivars.

The effective deployment of haplotype‐based breeding for the development of heat‐resilient rice cultivars requires the alignment of superior haplotypes with clearly defined breeding product profiles. For heat tolerance, these profiles include stable spikelet fertility under high temperatures (≥35°C during anthesis), maintenance of grain quality traits such as low chalkiness and stable amylose content under elevated temperatures, and consistent yield performance across diverse heat‐stress environments. Haplotype‐based approaches enable the identification of favorable allelic combinations across key genomic regions controlling complex traits, thereby facilitating more precise selection than single‐marker strategies (W. Wang, Mauleon, et al., 2018).

By integrating haplotype information with trait‐specific thresholds and multi‐environment phenotypic data, breeders can design targeted allelic combinations that confer both reproductive‐stage thermotolerance and grain quality stability. This strategy is particularly valuable for capturing the cumulative effects of linked loci underlying polygenic traits such as heat tolerance. In addition, recent advances in pangenomics and haplotype‐resolved assemblies have expanded the discovery of SVs and rare alleles associated with stress adaptation, further enhancing the utility of haplotype‐based breeding (P. Qin et al., 2021; Zhou et al., 2020). Aligning haplotype selection with breeding product profiles helps translate genomic variation into cultivars with clearly defined performance targets, thereby narrowing the gap between genetic discovery and field‐level deployment under climate variability.

### 7.5 Pangenome‐assisted discovery of novel SVs for heat tolerance

Pangenome analysis offers a comprehensive genomic approach that captures the entire gene repertoire of a species, encompassing both core genes shared by all individuals and accessory genes unique to specific lineages (D. Guo et al., 2025). This broader perspective overcomes the limitations of single‐reference genomes by revealing important genetic diversity hidden in non‐reference sequences. For example, the traditional Nipponbare reference genome represents only a small fraction of *O. sativa* diversity, omitting lineage‐specific genes found in *indica*, *aus*, *aromatic*, and wild AA‐genome relatives, many of which encode genes for stress‐response and adaptation (D. Guo et al., 2025; D. Wu et al., 2023; Q. Zhao, Feng, et al., 2018). Pangenome studies have shown that over 20% of rice genes are absent from the reference genome but are enriched for pathways involved in environmental adaptation and thermotolerance (D. Guo et al., 2025; Woldegiorgis et al., 2022; D. Wu et al., 2023; Yan et al., 2023). By integrating short‐ and long‐read sequencing, researchers have expanded the estimated rice genome size from approximately 374 Mb (Nipponbare) to 1.25–1.52 Gb, identifying nearly 10,000 dispensable genes enriched in stress and defense‐related pathways (Shang et al., 2022; F. Zhang, Xue, et al., 2022; Q. Zhao, Feng, et al., 2018). Some of these dispensable genes overlap with heat‐responsive loci identified in transcriptomic and QTL studies (Woldegiorgis et al., 2022). These findings make pangenomes powerful tools for discovering rare, population‐specific, or wild‐derived alleles that contribute to heat tolerance and were previously undetectable through reference‐based analyses.

Beyond expanding gene content, pangenome analysis also uncovers structural variation that shapes much of the genetic and regulatory diversity responsible for stress adaptation in rice. SVs including presence/absence variants (PAVs), copy‐number variations (CNVs), inversions, and TE‐associated rearrangements constitute a major component of pangenomic diversity and frequently underlie regulatory variation in stress‐response pathways (Jayakodi et al., 2025; Yan et al., 2023). Because stress adaptation in plants relies on the coordinated regulation of HSF–HSP modules, ROS signaling, and calcium‐mediated stress networks (Kan et al., 2023; X. Liu et al., 2025), mapping (SVs) is essential for identifying causal regulatory variants that are often missed by SNP‐only analyses. Recent advances in graph‐based pangenomes now enable more accurate SVs genotyping across diverse *Oryza* genomes and support SVs‐GWAS or graph‐anchored eQTL analyses that link SVs to stress‐inducible gene expression (D. Guo et al., 2025; Jayakodi et al., 2025). Moreover, SV‐derived markers demonstrate strong transferability and predictive potential across *indica*, *japonica*, and wild rice populations, making them highly effective tools for MAS, haplotype‐based breeding, and GS aimed at improving complex stress‐adaptive traits, including heat tolerance (D. Guo et al., 2025; Jayakodi et al., 2025).

Despite rapid advances in rice pangenomics, the effective integration of dispensable genes and SV‐based haplotypes into breeding pipelines remains limited. Pangenome analyses have revealed extensive PAV and structural variation, including insertions, deletions, and CNV, that contribute substantially to agronomic performance and environmental adaptation (Kou et al., 2020; P. Qin et al., 2021). These variants may influence trait expression through gene dosage effects, regulatory alterations, or the presence of novel genes absent from the reference genome. However, their direct deployment in breeding programs is constrained by the difficulty of translating complex genomic variation into breeder‐friendly markers and decision‐support frameworks.

In practical breeding pipelines, the operationalization of pangenome‐derived haplotypes involves a stepwise process: (i) identifying SV‐ and PAV‐based haplotypes associated with heat tolerance through pangenome‐wide analyses; (ii) converting these variants into robust, high‐throughput markers suitable for large breeding populations; (iii) validating haplotype effects across diverse environments and genetic backgrounds; and (iv) integrating them into GS models or MAS schemes for deployment in elite germplasm. Recent studies have shown that SV‐based markers and pangenome‐wide association approaches can identify causal variants often missed by SNP‐based analyses, thereby improving mapping resolution and selection efficiency (W. Wang, Mauleon, et al., 2018; Zhou et al., 2020).

The deployment of haplotypes derived from pangenome‐based analyses remains limited by challenges in large‐scale SV genotyping, computational complexity, and the lack of standardized SV‐aware breeding pipelines. In addition, the phenotypic effects of dispensable genes are often environment dependent, with strong genotype × environment interactions influencing their expression and contributions to stress adaptation. Addressing these constraints will require the integration of graph‐based genome frameworks, SV‐aware GS models, and haplotype‐informed prediction approaches. These advances will enable breeders to move beyond SNP‐centric selection and more fully exploit the functional diversity encoded by dispensable genomes and structural variation to develop heat‐resilient rice cultivars (Garrison et al., 2018).

Pangenome approaches further extend these insights by capturing SVs and PAVs that underlie genes discussed in earlier sections. Genes involved in reproductive development, stress signaling, and lipid metabolism may exhibit substantial haplotype diversity or structural variation across diverse germplasm. Integrating pangenome‐derived variation with GWAS signals and candidate genes enables more precise identification of functional alleles, thereby facilitating the deployment of superior haplotypes and SVs in breeding programs for heat tolerance (Zhou et al., 2020).

### 7.6 Genome editing for heat stress tolerance

The advent of CRISPR–Cas9 genome editing has revolutionized functional genomics and crop improvement by enabling precise and targeted modifications of stress‐responsive genes (Kumar et al., 2023). In the context of heat stress, this technology has been instrumental in dissecting regulatory pathways and engineering thermotolerant varieties (Chakraborty & Wylie, 2025; Kaur et al., 2025; Pandey et al., 2024; Shaheen et al., 2023). In rice, CRISPR/Cas9‐generated loss‐of‐function mutants of the membrane‐associated TF *OsNTL3* and the NAC factor *OsNAC006* exhibit pronounced heat sensitivity, demonstrating that these genes act as positive regulators of thermotolerance and linking UPR and ROS/homeostasis pathways to heat resilience (X.‐H. Liu, Lyu, et al., 2020; B. Wang, Zhong, et al., 2020). Building on this, targeted knockout of the NADPH oxidase *OsRbohB* via CRISPR/Cas reduced heat‐induced ROS overaccumulation, enhanced the expression of HSP/HSF genes, and improved grain yield under high‐temperature regimes, highlighting this gene as a promising target for breeding heat‐tolerant rice (X. Liu et al., 2025). Beyond rice, CRISPR/Cas9‐mediated disruption of *SlMAPK3* in tomato (*Solanum lycopersicum* L.) confers enhanced thermotolerance through reduced oxidative damage and elevated antioxidant and HSF/HSP activity (W. Yu et al., 2019). Similarly, genome‐edited heat‐responsive HSFs in *Arabidopsis* illustrate how editing can resolve positive versus negative regulators within heat shock networks (Vu et al., 2023; Wenjing et al., 2020). Emerging multiplex editing and CRISPR activation (CRISPRa) platforms now enable simultaneous tuning of key transcriptional hubs (e.g., HSFs, NACs, UPR components) and signaling nodes, offering a rational route to stack beneficial alleles and fine‐tune inducible protection under heat stress (Chakraborty & Wylie, 2025; Kaur et al., 2025; Vu et al., 2023). Collectively, these advances position CRISPR–Cas systems as powerful tools for both validating components of plant heat stress networks and engineering climate‐resilient cultivars through targeted rewiring of their regulatory circuitry.

From a translational perspective, the deployment of genome editing in rice is shaped not only by technical advances but also by regulatory frameworks that differ across major rice‐producing regions. Countries such as the United States and several others have adopted product‐based regulatory approaches under which genome‐edited crops lacking foreign DNA insertions may be exempt from stringent GMO regulations, thereby facilitating field testing and commercialization. In contrast, the European Union currently regulates most genome‐edited crops under GMO legislation, which may constrain their deployment despite the precision of these edits (Jaganathan et al., 2018; Wolt et al., 2016). Several Asian countries, including China, Japan, and India, are also developing regulatory pathways for genome‐edited crops, with growing acceptance of non‐transgenic edits creating new opportunities for rice improvement programs.

Beyond CRISPR/Cas9‐mediated gene knockouts, emerging genome‐editing platforms such as base editing and prime editing are broadening the scope of precise genetic modification in rice. Base editors enable targeted single‐nucleotide substitutions without introducing double‐strand breaks, thereby allowing precise modification of alleles associated with stress tolerance and grain quality (Zong et al., 2017). Prime editing further extends this capability by enabling targeted insertions, deletions, and all possible base‐to‐base conversions without donor templates, providing a versatile platform for fine‐tuning gene function (Anzalone et al., 2019). Although these technologies continue to be optimized for efficiency and delivery in rice, they hold substantial promise for accelerating the development of heat‐resilient cultivars through precise and predictable genome modification.

### 7.7 Agronomic practices to alleviate heat‐induced damage

A variety of agronomic strategies have been successfully employed to mitigate heat‐induced damage in rice. The application of specific plant growth regulators, including CTKs, SA, BRs, and ethylene‐releasing agents can alleviate key effects of heat stress such as pollen sterility, reduced spikelet formation, lower grain weight, and decreased seed set. In addition, compounds like polyamines, osmoprotectants, and antioxidants play important role in stress mitigation (Alcázar et al., 2011). For example, endogenous ascorbic acid helps regulate ROS levels, preserving leaf health during heat exposure (Q. L. Zhang, Wei, et al., 2018). The external application of spermidine enhances antioxidant defenses and photosynthetic performance, thereby improving yield stability under thermal stress. Similarly, protective agents such as glycine betaine and proline safeguard cellular structures and support Rubisco activity, helping maintain productivity under high temperatures (Fahad et al., 2016). Nutrient management strategies also contribute to heat stress resilience. Moderate nitrogen supplementation, combined with phosphorus and biochar, has been shown to reduce yield loss during the reproductive phase (Ali et al., 2020). Additionally, mist irrigation during flowering serves as an effective cooling technique, lowering ambient temperatures, delaying leaf aging, and enhancing antioxidant enzyme activity, all of which help mitigate yield declines caused by heat (Jiang et al., 2020).

## 8 CONCLUSIONS AND FUTURE DIRECTIONS

As global temperatures continue to rise, heat stress has become a major constraint on rice production by disrupting key physiological, biochemical, and molecular processes. Rice is particularly vulnerable during the reproductive stage, when elevated temperatures reduce pollen viability, increase spikelet sterility, and ultimately compromise grain yield and grain quality. Heat stress also impairs photosynthetic efficiency, membrane stability, carbohydrate metabolism, and phytohormone balance, thereby exacerbating yield losses. Consequently, the development of thermotolerant rice cultivars is critical for sustaining rice productivity and safeguarding global food security. Recent advances in the understanding of heat stress responses, particularly heat shock factor‐mediated transcriptional regulation, ROS and lipid signaling, and epigenetic regulation, have substantially expanded current knowledge of thermotolerance mechanisms. In addition, the identification of heat‐tolerance‐associated QTLs and the characterization of key regulatory genes such as *OsHTAS*, *SLG1*, *Sus3*, and *TT1* have provided valuable targets for functional validation and genomics‐assisted breeding.

The development of heat‐resilient rice requires an integrated strategy that combines genomic, agronomic, and computational innovations. GWAS, GS, and CRISPR/Cas9 genome editing can accelerate the identification and deployment of favorable alleles, whereas pyramiding stable nonallelic QTLs may enhance resilience across diverse environments. Agronomic interventions such as optimized nutrient management, precision irrigation, foliar applications, and exogenous treatment with CTKs, BRs, and polyamines can further mitigate heat‐induced damage by strengthening antioxidant defenses and protecting cellular integrity. Early morning flowering also represents a promising adaptative trait because it shifts anthesis to cooler morning hours, thereby reducing heat‐induced sterility in reproductive tissues. Emerging evidence further highlights the importance of epigenetic regulation and small RNA‐mediated heat stress memory, with chromatin modifications and miRNAs such as miR398 and miR156 playing important roles in modulating transcriptional responses during recurrent heat exposure.

Recent advances in artificial intelligence (AI) and high‐throughput phenotyping technologies are transforming the evaluation of heat stress responses in rice. Imaging‐based phenomics approaches, including hyperspectral, thermal, RGB, and UAV‐based imaging, enable rapid and nondestructive assessment of physiological, biochemical, and reproductive traits under field conditions. These tools support large‐scale screening of breeding populations and provide accurate estimates of traits such as canopy temperature, transpiration efficiency, pollen viability, and spikelet fertility under heat stress. In parallel, machine learning and deep learning algorithms facilitate the analysis of complex phenotypic datasets and improve stress classification and performance prediction across environments. The integration of AI‐driven phenotyping with GS and GWAS provides a powerful framework for linking genotype to phenotype, accelerating breeding cycles, and identifying heat‐resilient genotypes. Collectively, the integration of molecular genetics, precision phenotyping, agronomic management, and AI‐assisted breeding offers a promising pathway for developing climate‐resilient rice cultivars capable of sustaining productivity under intensifying global warming.

## AUTHOR CONTRIBUTIONS

**Prabhat Rana**: Writing—original draft; writing—review and editing. **Chanderkant Chaudhary**: Writing—review and editing. **Rajat Pruthi**: Writing—review and editing. **Mansi Sharma**: Writing—review and editing. **Bhupinderjeet Singh**: Data curation; writing—original draft; writing—review and editing. **Ravi Kiran Reddy Kondi**: Writing—review and editing. **Mallesham Bulle**: Writing—review and editing. **Prasanta K. Subudhi**: Conceptualization; funding acquisition; project administration; supervision; writing—review and editing.

## CONFLICT OF INTEREST STATEMENT

The authors declare no conflicts of interest.

## Data Availability

There are no original data associated with this article. Referenced data are available in the literature.
